# Supplementary material for: Collective communication and behaviour in response to uncertain ‘Danger’ in network experiments
Source: Proc Math Phys Eng Sci. 2020 May 27;476(2237):20190685. doi: 10.1098/rspa.2019.0685 (PMC7277132; doi:10.1098/rspa.2019.0685)
Supplement: Supplementary Material for Collective Communication and Behavior in Response to Uncertain “Danger” in Network Experiments [file rspa20190685supp1.pdf]

Supplementary Material

for

Collective Communication and Behavior in Response to Uncertain "Danger" in Network Experiments

Hirokazu Shirado[1], Forrest W. Crawford[2,3], Nicholas A. Christakis[2,4]

[1] *School of Computer Science, Carnegie Mellon University, Pittsburgh PA 15213 USA.*

[2] *Yale Institute for Network Science, Yale University, New Haven, Connecticut 06520, USA.*

[3] *Department of Biostatistics, Yale School of Public Health, New Haven, CT 06510, USA.*

[4] *Department of Sociology, Yale University, New Haven, Connecticut 06520, USA.*

Supplementary Figures 2

Supplementary Tables 11

Supplementary Methods 14

Instructions and tutorial 14

Modeling of expected utility for sequential decision-making 40

Analysis of supplementary sessions with information availability after evacuation 41

Analysis of supplementary sessions with other network structures 43

Supplementary Figures

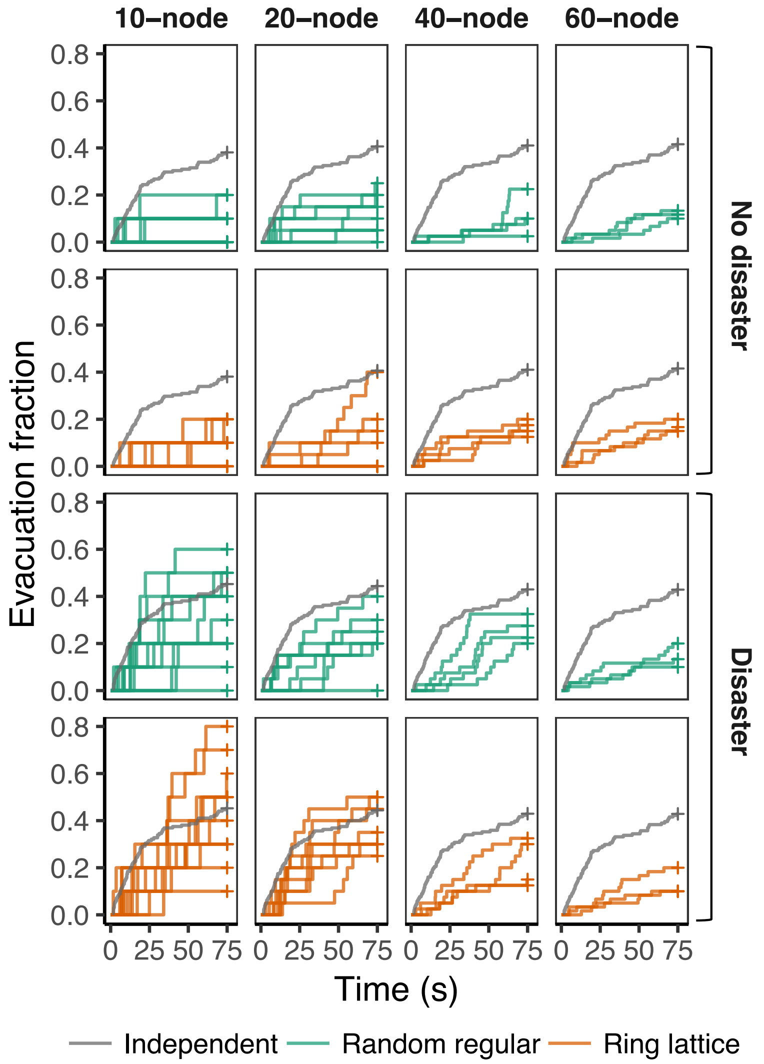

Figure S1

Each session's evacuation fractions over time. Color lines show results for each network session (*N*=12 for 10-node; *N*=8 for 20-node; *N*=4 for 40-node; *N*=3 for 60-node networks), by their network topology (line color), network size (horizontal dimension), and disaster condition (vertical dimension). Grey lines show results of the independent condition, which are identical with the exception of the single informed subject (who differed in the disaster and no-disaster situations).

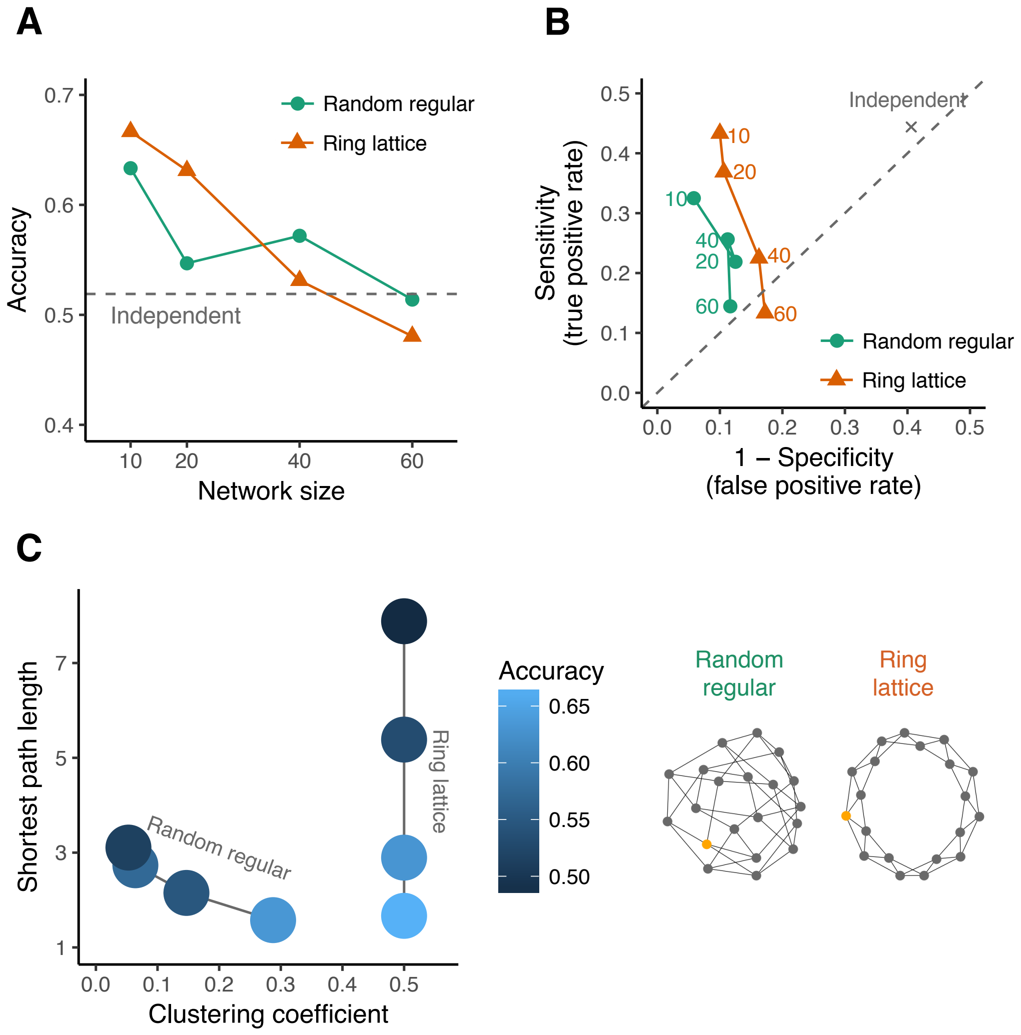

Figure S2

Performance of binary classification tests of evacuation game by network treatments. Sensitivity measures the proportion of positives (disaster) that are correctly identified (evacuation). Specificity measures the proportion of negatives (no disaster) that are correctly identified (staying) (i.e., Sensitivity is true positive rate; Specificity is true negative rate; and 1 – Specificity is false positive rate). Accuracy measures the proportion of overall correct identification. All the parameters are calculated with individual decisions at the end of session (75 seconds). (A) Accuracy over network type and size. The dashed line indicates the accuracy of the independent condition (= 0.519). (B) Receiver operating characteristic (ROC) curves of social predictors of disaster. The annotated numbers indicate network size. As the network size increases, the group-level sensitivity of disaster prediction improves from the level of random guess. (C) Accuracy by graph attributes. Few clusters and long average path length reduce the accuracy of disaster prediction.

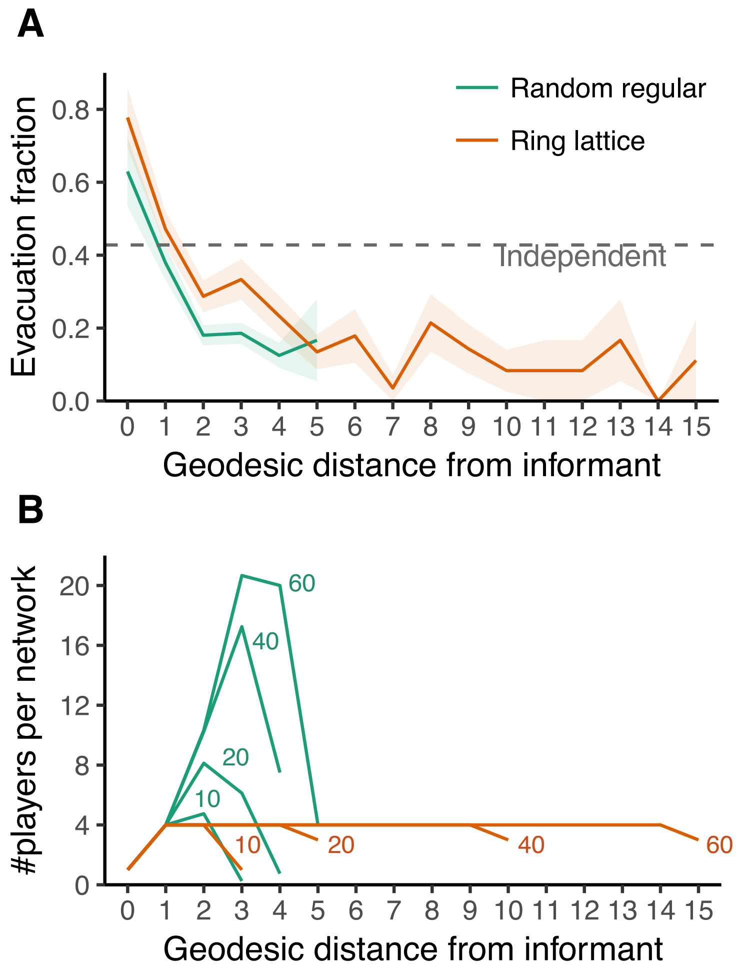

Figure S3

Evacuation performance by geodesic distance from informant. (A) Evacuation fraction at the end of session (75 sec) by geodesic distance from an informant in the disaster situation, across network type. Shades are standard error among sessions. Dashed line indicates the evacuation fraction of the independent condition. (B) Number of players per network by geodesic distance from an informant, across network type. The annotated numbers indicate network size. The informant's effect explains the structural difference in the diffusion of evacuation behavior. On the one hand, players in a ring-lattice network were more likely to evacuate than those who were located at the same geodesic distance from an informant in a random-regular network. The evacuation fraction was 20% or less in the players who were 2 hops and further away from an informant in a random regular network or 5 hops and further away from an informant in a ring-lattice network. On the other hand, random-regular networks have many players within a short range from an informant, compared to ring-lattice networks.

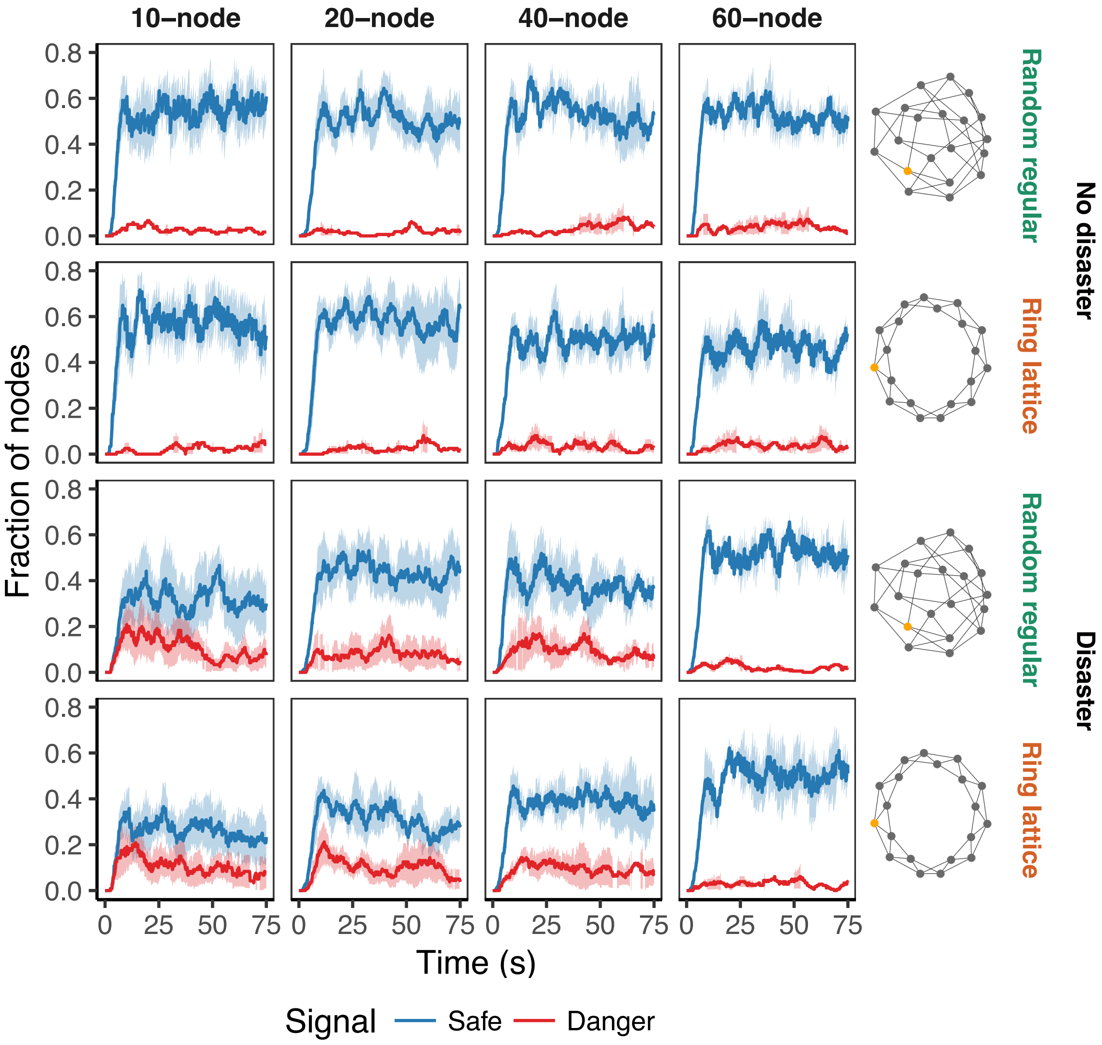

Figure S4

Fraction of signals over time, across network topology and size. Average fraction of nodes showing safe (blue lines), danger signals (red lines), and evacuations (green lines) over time. Shades are 95% confidential intervals among network sessions (*N*=12 for 10-node; *N*=8 for 20-node; *N*=4 for 40-node; *N*=3 for 60-node networks). The network snapshots are 20-node networks.

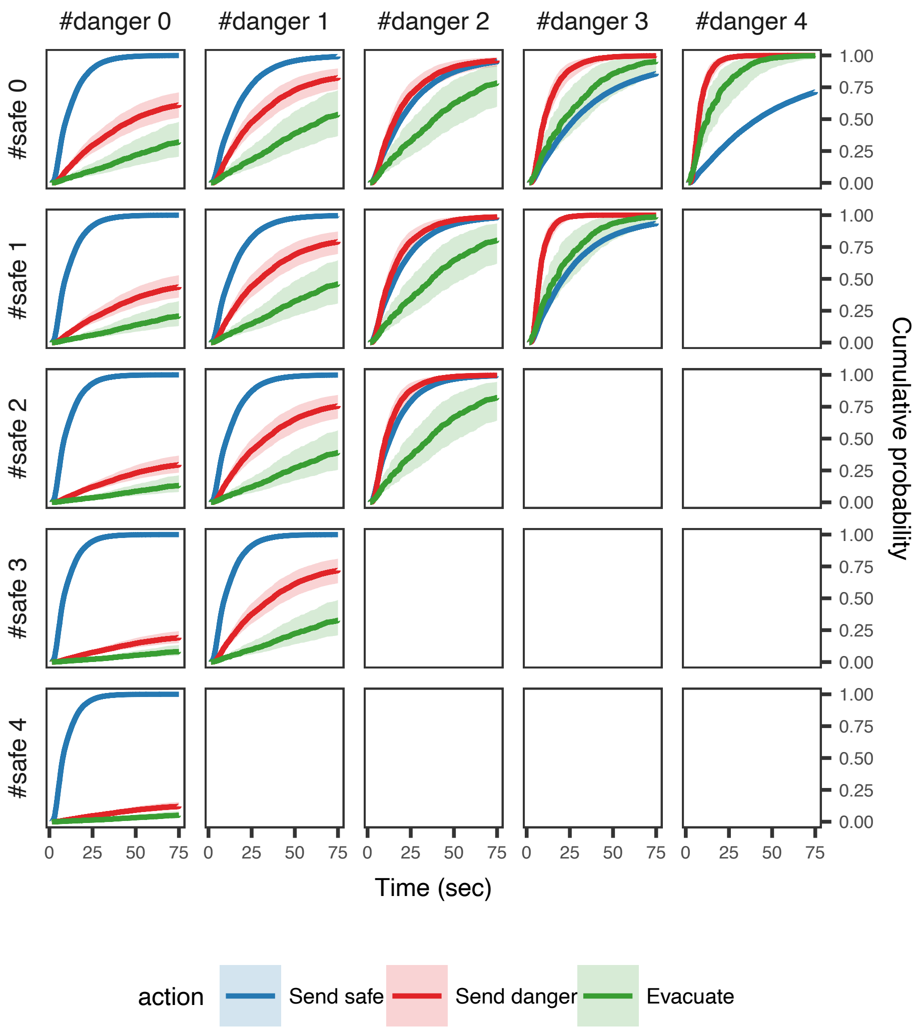

Figure S5

Full version of estimated cumulative probability of subject's action over time, across signal exposure from neighbors. Error bars are 95% confidence intervals. Figure 4C shows the cells of 0, 2, and 4 safe and danger signals in this figure. The results suggest the social conditions that are superior for warnings to spread better than safe messages and hence to (properly) promote evacuation (i.e., the right direction in Fig. 4C and figure S5). Subjects strongly follow their local neighbors regarding whether to give warnings, in that the hazard of sending a danger signal increases 1.84 times (95% CI: 1.72 – 1.98) with an additional danger signal in neighbors, while controlling for other types of signal exposure; on the other hand, that of sending a safe signal increases only 1.09 times (95% CI: 1.07 – 1.11) with an additional safe signal in neighbors (Fig. 4B). Only when the number of warning neighbors exceeds that of reassuring neighbors is a subject more likely to send a danger signal than a safe one. Likewise, subjects need more warnings than safe messages to decide to evacuate.

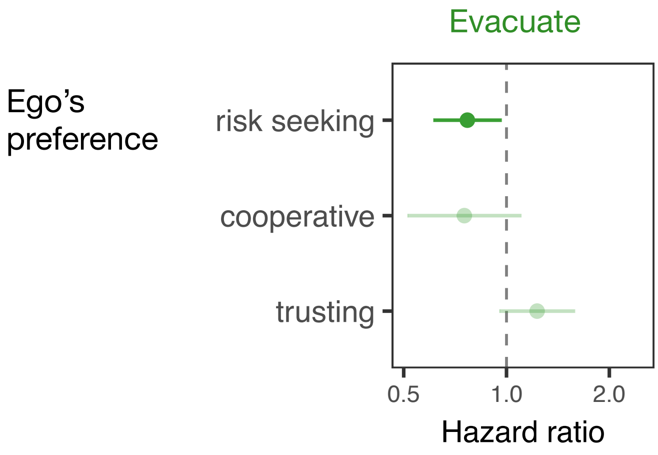

Figure S6
Estimated hazard ratios of evacuation related to personal preferences (risk, cooperation, and trust) in the independent condition. These are estimated by Cox proportional models for the sessions in the independent condition. Error bars are 95% confidence intervals. Dark color indicates significant coefficients.

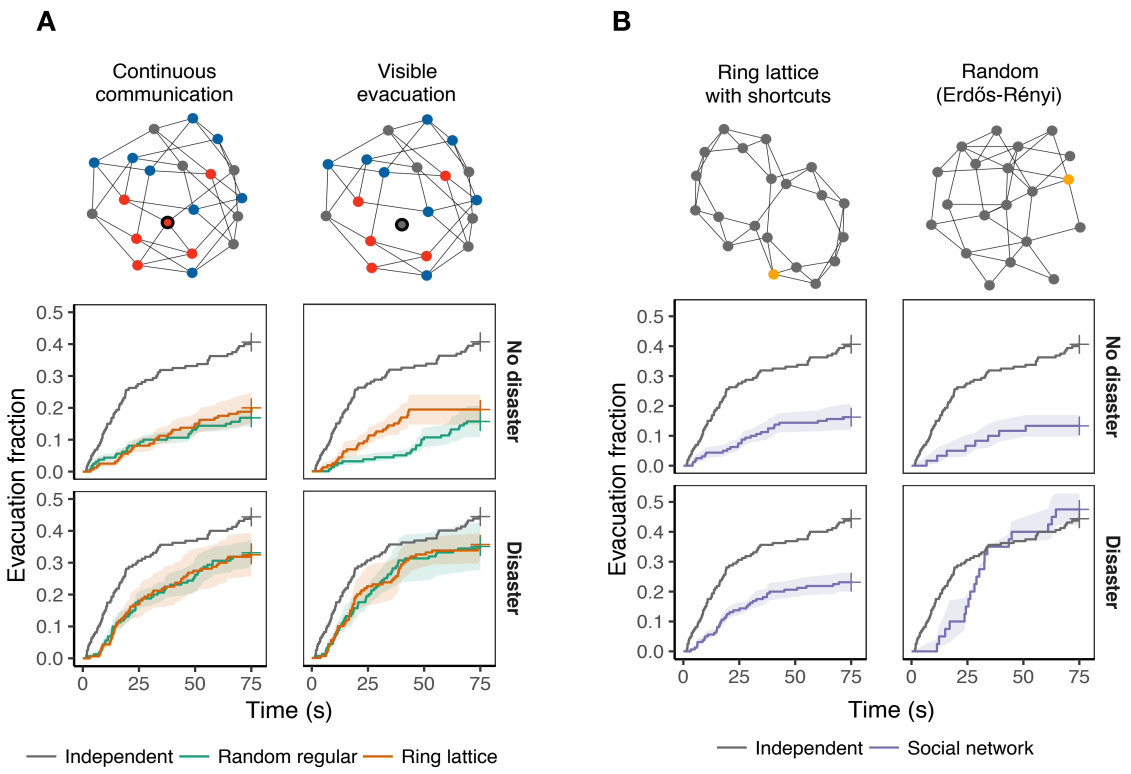

Figure S7

Evacuation diffusion with additional information availability and network structural heterogeneity. (A) Lines indicate average fractions of evacuated subjects over time. Shades are standard error among network sessions (*N*=8). The lines of the independent condition are identical with the exception of informed subjects. In the continuous communication condition, subjects could send a signal to neighbors even after they evacuated. In the visible evacuation condition, the node and links of an evacuated subject were removed in a network. That is, subjects in play could observe their neighbors' evacuation. All the networks had 20 nodes. (B) Lines indicate average fractions of evacuated subjects over time. Shades are standard error among network sessions (*N*=8 for ring lattice with shortcuts without disaster, *N*=8 for ring lattice with shortcuts with disaster, *N*=3 for Erdős-Rényi random network without disaster, N=2 for Erdős-Rényi random network with disaster). The lines of the independent condition are identical with the exception of informed subjects. Ring lattice networks with shortcuts were created by rewiring an opposite link pair of a ring lattice. Erdős-Rényi random networks had heterogeneous degree distribution with the same number of edges as other types of network. An informed subject was randomly selected in a network. Thus, the informant had different number of neighbors by chance in an Erdős-Rényi random network. All the networks had 20 nodes.

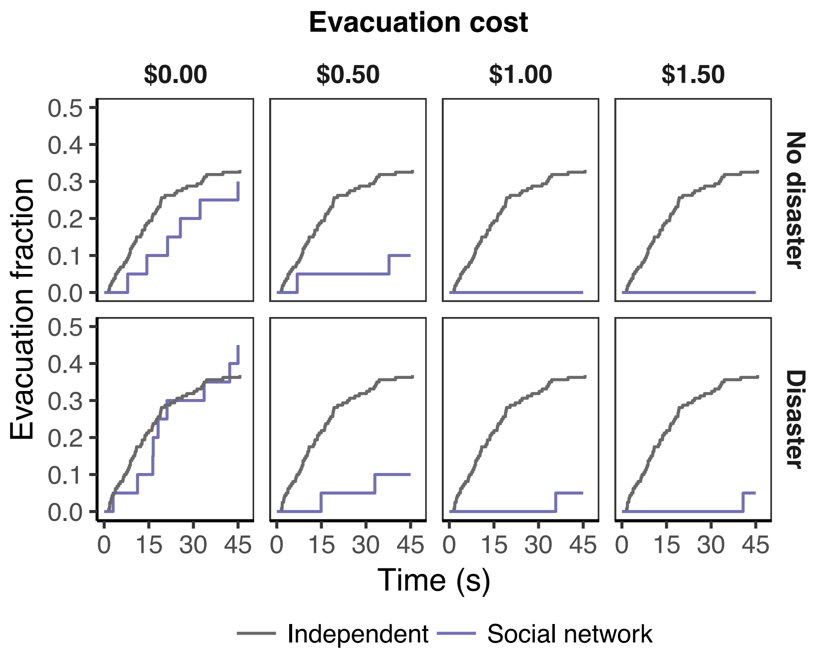

Figure S8

Evacuation diffusion according to evacuation cost. Lines indicate fractions of evacuated subjects over time. Each treatment has a session. We tested the impact of varying the evacuation cost with a session for each treatment combination: four levels of evacuation cost ($0.00, $0.50, $1.00, and $1.50) crossed with whether a disaster would strike. In this test, subjects played the evacuation game in an Erdős-Rényi random network with 20 nodes and density=0.20. In contrast of the main experiment, subjects were not informed about which node was an informed subject even when they were connected to the informant. Also, the game ended in 45 seconds. For comparison, the lines of the independent condition are shown. The results show that a modest cost was sufficient to keep subjects from evacuating in the social network condition. Only when subjects could evacuate without any cost did they evacuate from a disaster at the same level as the independent condition where subjects paid $1 for evacuation. A reduction in evacuation cost promoted successful evacuation from a disaster, but, at the same time, it increased needless evacuations. These results suggest that the change in payoff structure does not improve the accuracy of collective prediction in networked groups.

A. Total time period for informants to show safe signals

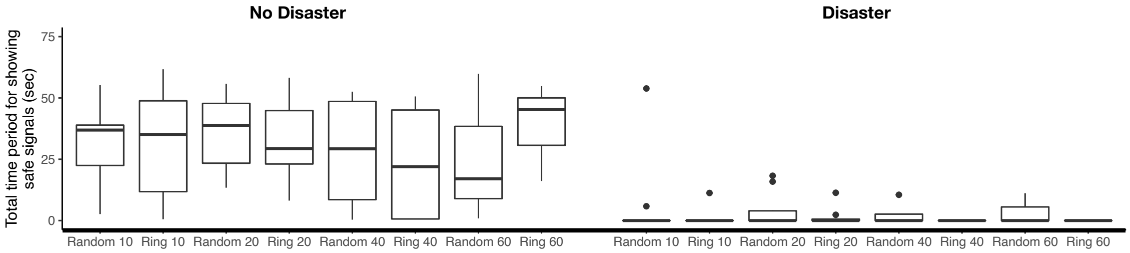

B. Total time period for informants to show danger signals

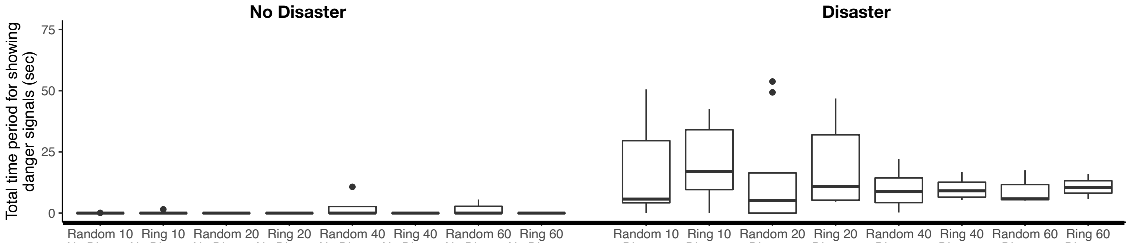

Figure S9

Total time period for informants to send signals. The information quantity of signals from informants varies with whether a disaster strikes, but it does not vary significantly across the network treatments. It should be noted that informants could change their behaviors depending on their neighbors' behaviors. For example, informants might spend a longer time to send certain signals when the neighbors did not copy their signals.

Supplementary Tables

Table S1

The results of statistical analysis regarding whether to evacuate, by group-level treatments, estimated by Cox proportional hazard model incorporating random effect for session. *N*=1,400 for each model. The reference category is the independent condition.

A. No disaster

Network type	Network size	Hazard ratio	95% C.I.	P value
Independent	-	-	-	-
Random regular	10	0.127	(0.060 - 0.268)	< 0.001
Random regular	20	0.245	(0.145 - 0.413)	< 0.001
Random regular	40	0.212	(0.121 - 0.372)	< 0.001
Random regular	60	0.224	(0.130 - 0.386)	< 0.001
Ring lattice	10	0.190	(0.102 - 0.357)	< 0.001
Ring lattice	20	0.202	(0.116 - 0.352)	< 0.001
Ring lattice	40	0.323	(0.196 - 0.532)	< 0.001
Ring lattice	60	0.345	(0.212 - 0.563)	< 0.001

B. Disaster

Network type	Network size	Hazard ratio	95% C.I.	P value
Independent	-	-	-	-
Random regular	10	0.632	(0.421 - 0.947)	0.026
Random regular	20	0.391	(0.255 - 0.598)	< 0.001
Random regular	40	0.456	(0.298 - 0.697)	< 0.001
Random regular	60	0.245	(0.149 - 0.402)	< 0.001
Ring lattice	10	0.873	(0.601 - 1.268)	0.470
Ring lattice	20	0.734	(0.508 - 1.061)	0.100
Ring lattice	40	0.396	(0.255 - 0.615)	< 0.001
Ring lattice	60	0.225	(0.136 - 0.374)	< 0.001

Table S2

The results of statistical analysis regarding whether to evacuate and whether to send a signal (safe/danger), estimated by Cox proportional model with time-varying covariates of neighbor's signals and subject's past actions incorporating the random effect for individuals. The models control the repeat count of subject's past signaling. Figure 4B shows the estimated hazard ratios.

A. Evacuation

		Hazard ratio	95% C.I.	P value
Eposure from alters	#safe	0.602	(0.529 - 0.684)	< 0.001
	#danger	1.976	(1.716 - 2.275)	< 0.001
	#safe : #danger	1.336	(1.173 - 1.523)	< 0.001
Ego's past actions	#sending safe	0.940	(0.901 - 0.973)	< 0.001
	#sending danger	1.157	(1.091 - 1.226)	< 0.001
Network property	network size	0.996	(0.989 - 1.002)	0.170
	network type (ref.= random regular)	1.199	(0.957 - 1.502)	0.120
Ego's preference	risk-seeking score	0.997	(0.795 - 1.250)	0.980
	cooperative score	1.021	(0.832 - 1.251)	0.840
	trusting score	0.996	(0.788 - 1.259)	0.970

B. Sending a safe signal

		Hazard ratio	95% C.I.	P value
Eposure from alters	#safe	1.088	(1.071 - 1.105)	< 0.001
	#danger	0.629	(0.587 - 0.674)	< 0.001
	#safe : #danger	1.099	(1.051 - 1.150)	< 0.001
Ego's past actions	#sending safe	1.015	(1.013- 1.016)	< 0.001
	#sending danger	0.973	(0.960 - 0.985)	< 0.001
Network property	network size	1.001	(0.999 - 1.003)	0.210
	network type (ref.= random regular)	0.997	(0.934 - 1.064)	0.920
Ego's preference	risk-seeking score	1.132	(1.058 - 1.212)	< 0.001
	cooperative score	0.945	(0.889 - 1.005)	0.072
	trusting score	1.035	(0.967 - 1.109)	0.320

C. Sending a danger signal

		Hazard ratio	95% C.I.	P value
Eposure from alters	#safe	0.608	(0.568 - 0.651)	< 0.001
	#danger	1.837	(1.711 - 1.972)	< 0.001
	#safe : #danger	1.466	(1.376 - 1.562)	< 0.001
Ego's past actions	#sending safe	0.996	(0.985 - 1.006)	0.420
	#sending danger	1.198	(1.179 - 1.216)	< 0.001
Network property	network size	0.996	(0.992 - 1.000)	0.062
	network type (ref.= random regular)	0.955	(0.832 - 1.097)	0.520
Ego's preference	risk-seeking score	1.030	(0.894 -1.185)	0.690
	cooperative score	0.937	(0.826 - 1.062)	0.310
	trusting score	0.954	(0.825 - 1.104)	0.530

Table S3

The results of statistical analysis regarding whether to evacuate and whether to send a signal (safe/danger), estimated by Cox proportional model with time-varying covariates of neighbor's signals incorporating the random effect for individuals. In contrast of Table S2, the models do not include the repeat count of subject's past signaling. The estimation results are similar to those of the original models.

A. Evacuation

		Hazard ratio	95% C.I.	P value
Eposure from alters	#safe	0.580	(0.513 - 0.655)	< 0.001
	#danger	1.981	(1.748 - 2.245)	< 0.001
	#safe : #danger	1.342	(1.187 - 1.518)	< 0.001
Network property	network size	0.995	(0.990 - 1.001)	0.090
	network type (ref.= random regular)	1.147	(0.945 - 1.391)	0.170
Ego's preference	risk-seeking score	1.002	(0.829 - 1.210)	0.990
	cooperative score	0.990	(0.836 - 1.172)	0.910
	trusting score	0.995	(0.817 - 1.210)	0.960

B. Sending a safe signal

		Hazard ratio	95% C.I.	P value
Eposure from alters	#safe	1.088	(1.071 - 1.106)	< 0.001
	#danger	0.625	(0.583 - 0.676)	< 0.001
	#safe : #danger	1.094	(1.046 - 1.145)	< 0.001
Network property	network size	1.002	(1.000 - 1.005)	0.042
	network type (ref.= random regular)	1.039	(0.963 - 1.121)	0.320
Ego's preference	risk-seeking score	1.169	(1.080 - 1.266)	< 0.001
	cooperative score	0.933	(0.868 - 1.003)	0.059
	trusting score	1.045	(0.964 - 1.132)	0.290

C. Sending a danger signal

		Hazard ratio	95% C.I.	P value
Eposure from alters	#safe	0.587	(0.547 - 0.629)	< 0.001
	#danger	1.745	(1.623 - 1.876)	< 0.001
	#safe : #danger	1.453	(1.362 - 1.549)	< 0.001
Network property	network size	0.993	(0.988 - 0.998)	0.004
	network type (ref.= random regular)	1.029	(0.867 - 1.222)	0.740
Ego's preference	risk-seeking score	0.992	(0.832 -1.185)	0.930
	cooperative score	0.885	(0.758 - 1.033)	0.120
	trusting score	0.910	(0.758 - 1.093)	0.310

Supplementary Methods

Instructions and tutorial

Below are screenshots for the initial description of the tutorial and the confirmation tests. We also show the example screenshots of the real game.

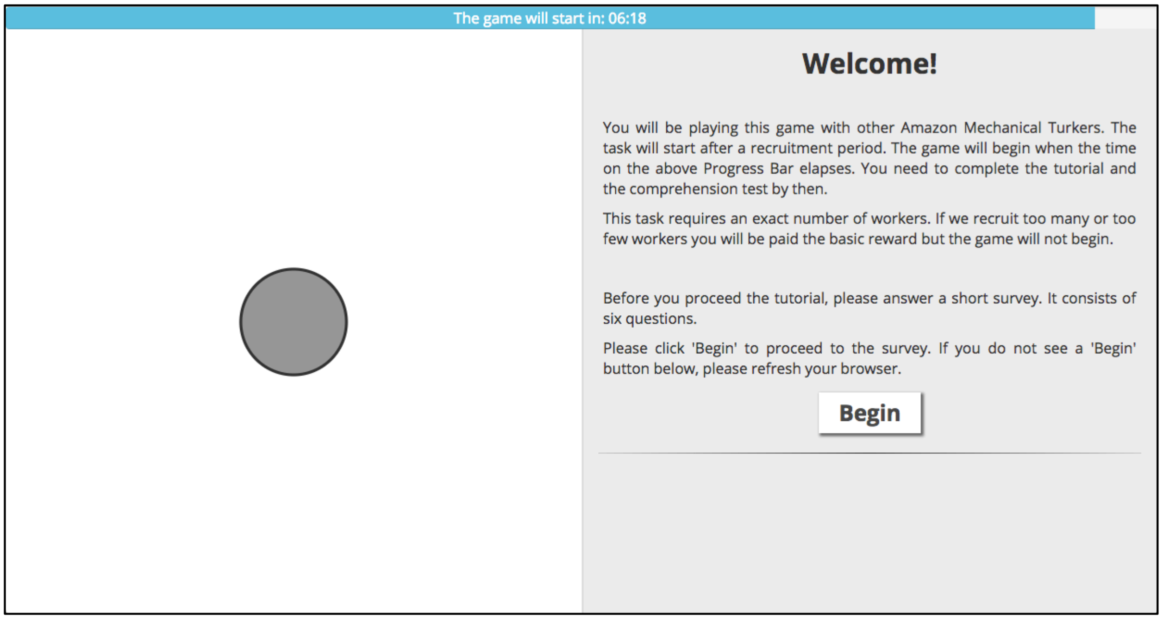

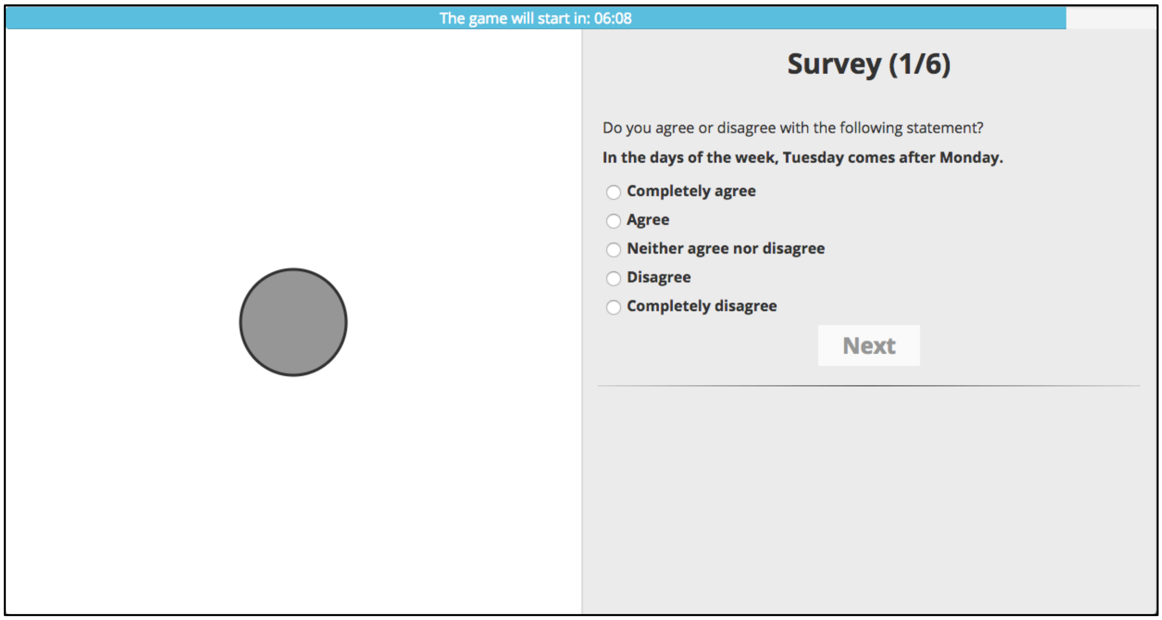

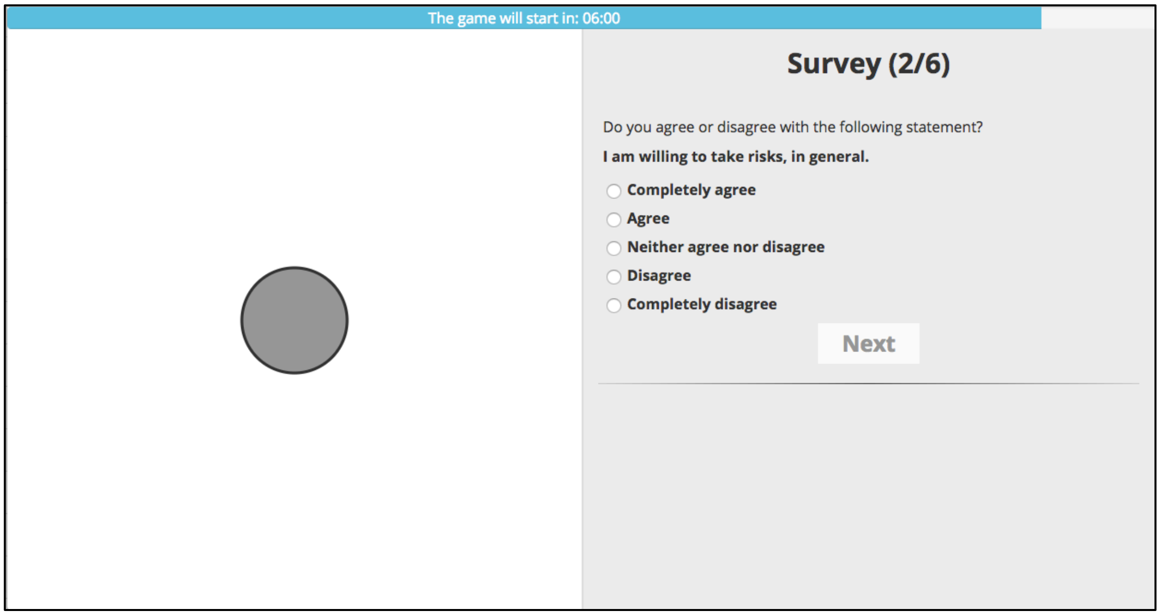
The game will start in: 06:00
Survey (2/6)
Do you agree or disagree with the following statement?
I am willing to take risks, in general.
Completely agree
Agree
Neither agree nor disagree
Disagree
Completely disagree
Next

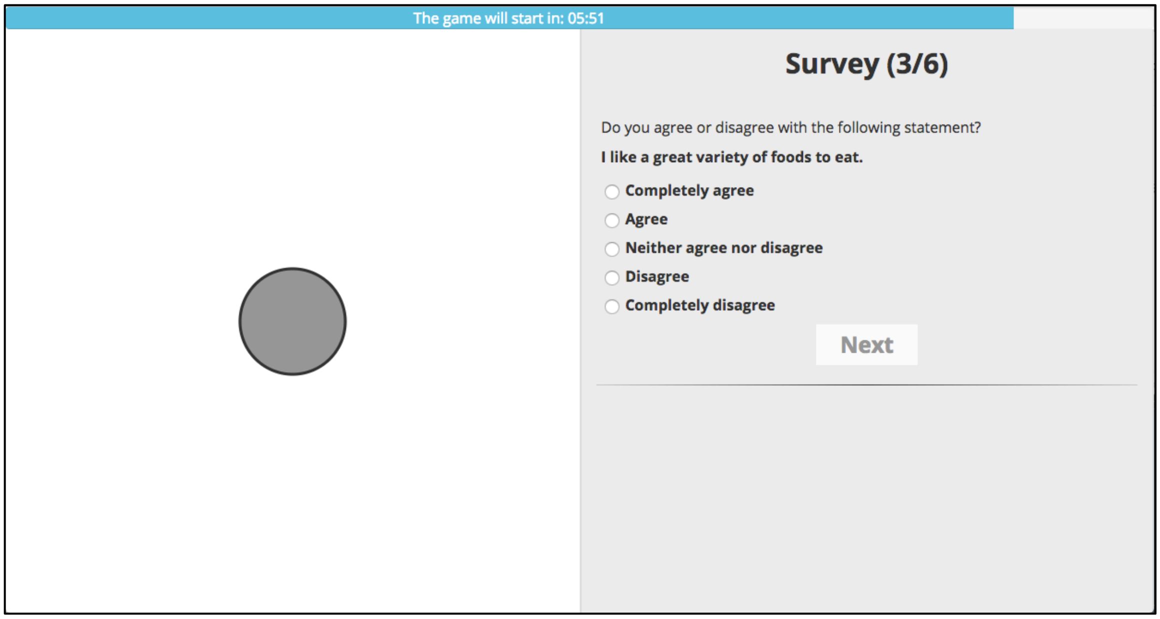
The game will start in: 05:51
Survey (3/6)
Do you agree or disagree with the following statement?
I like a great variety of foods to eat.
Completely agree
Agree
Neither agree nor disagree
Disagree
Completely disagree
Next

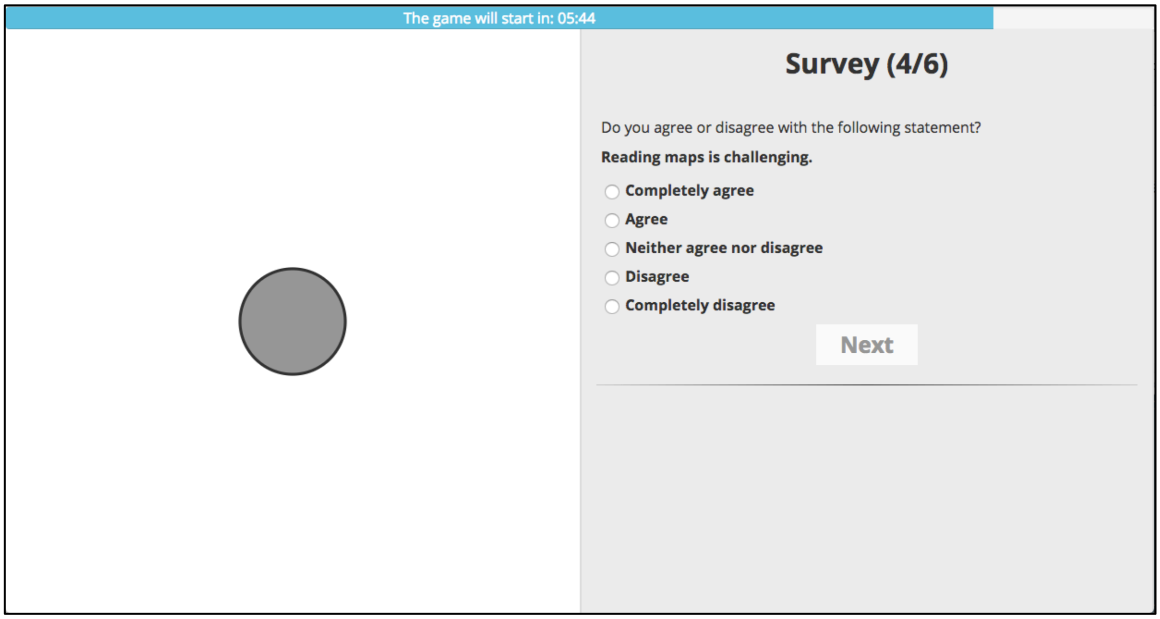
The game will start in: 05:44
Survey (4/6)
Do you agree or disagree with the following statement?
Reading maps is challenging.
Completely agree
Agree
Neither agree nor disagree
Disagree
Completely disagree
Next

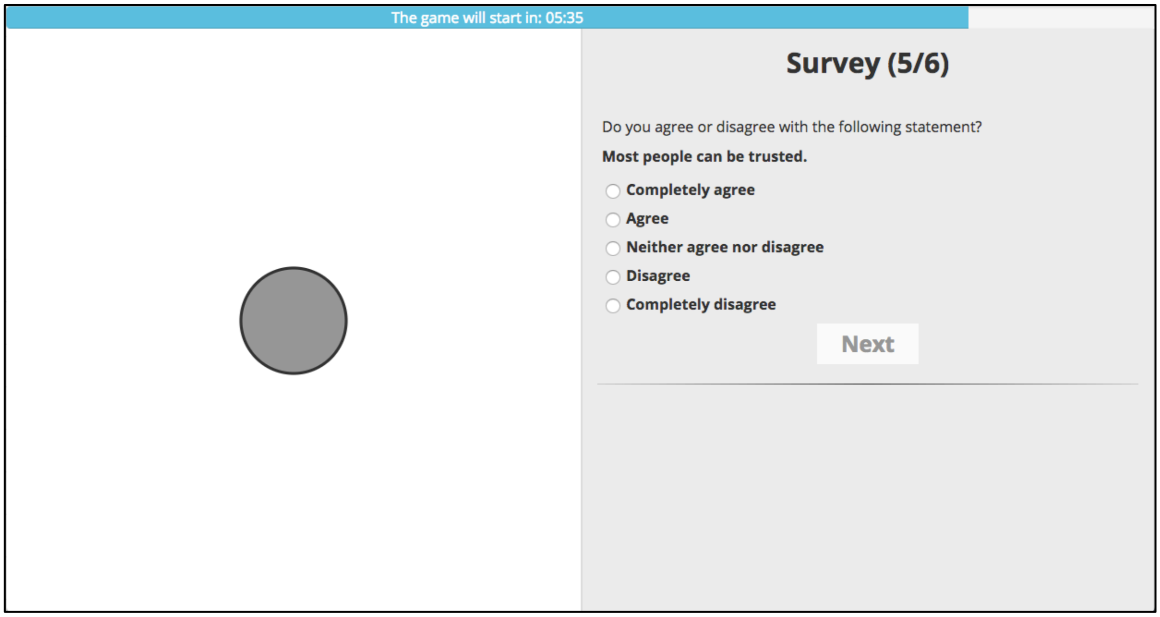
The game will start in: 05:35
Survey (5/6)
Do you agree or disagree with the following statement?
Most people can be trusted.
Completely agree
Agree
Neither agree nor disagree
Disagree
Completely disagree
Next

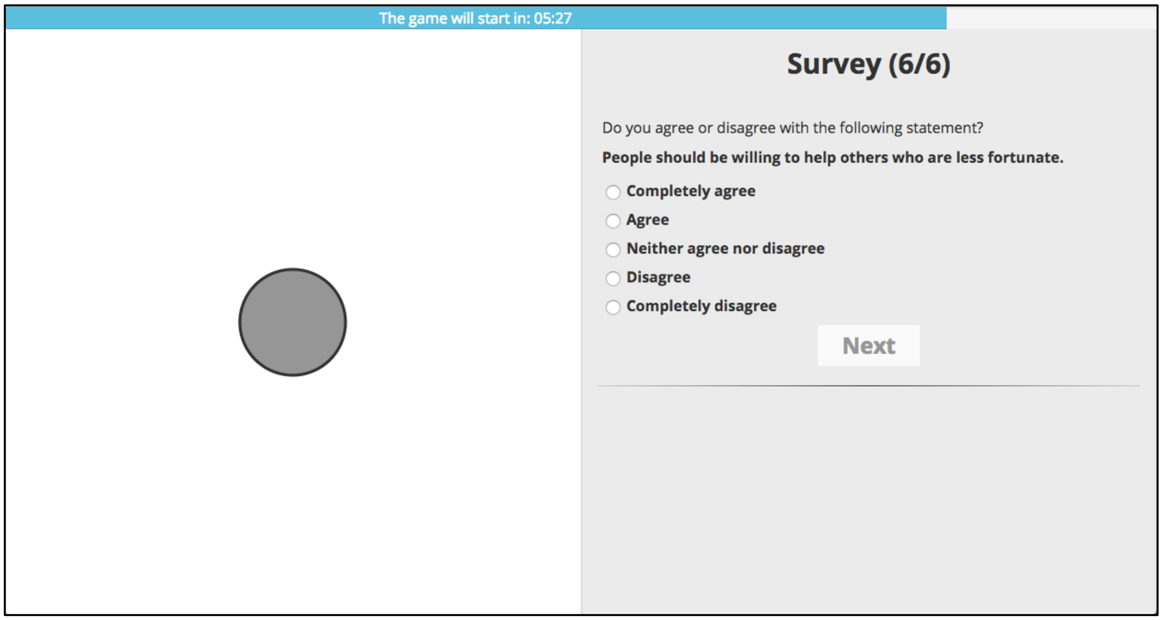
The game will start in: 05:27
Survey (6/6)
Do you agree or disagree with the following statement?
People should be willing to help others who are less fortunate.
Completely agree
Agree
Neither agree nor disagree
Disagree
Completely disagree
Next

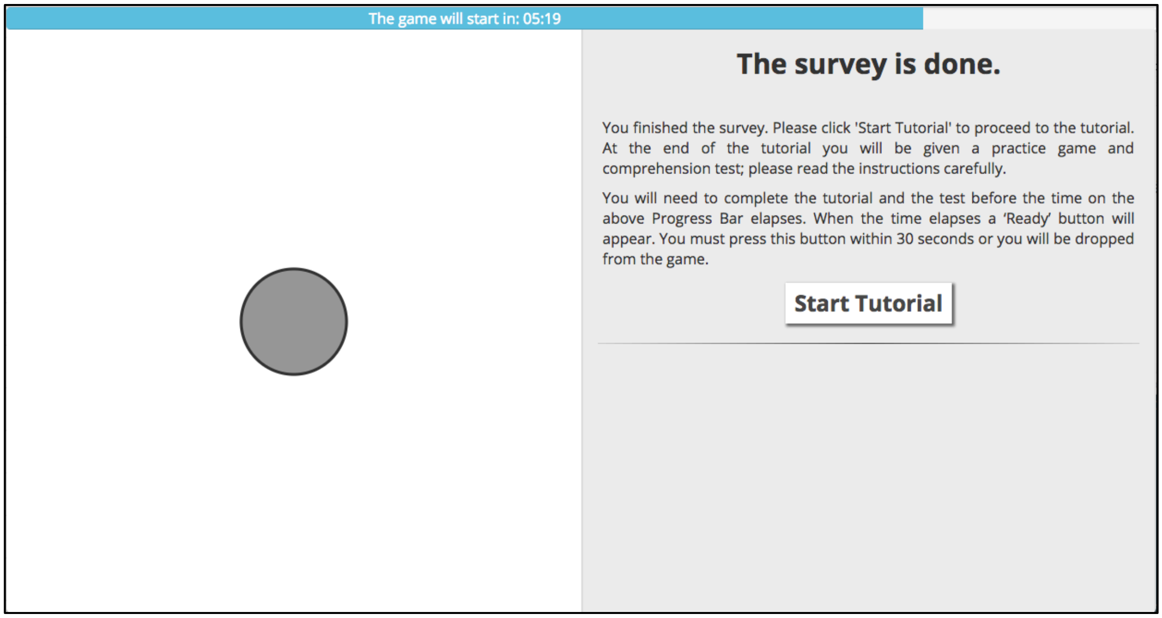
The game will start in: 05:19
The survey is done.
You finished the survey. Please click 'Start Tutorial' to proceed to the tutorial. At the end of the tutorial you will be given a practice game and comprehension test; please read the instructions carefully.
You will need to complete the tutorial and the test before the time on the above Progress Bar elapses. When the time elapses a 'Ready' button will appear. You must press this button within 30 seconds or you will be dropped from the game.
Start Tutorial

A. Social network condition

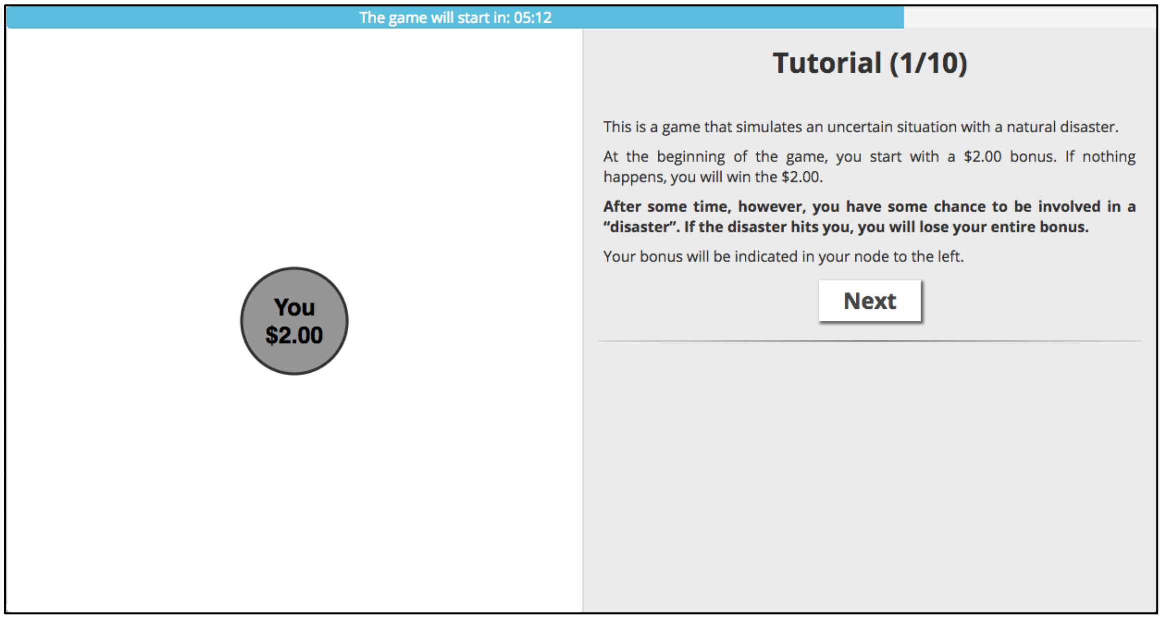

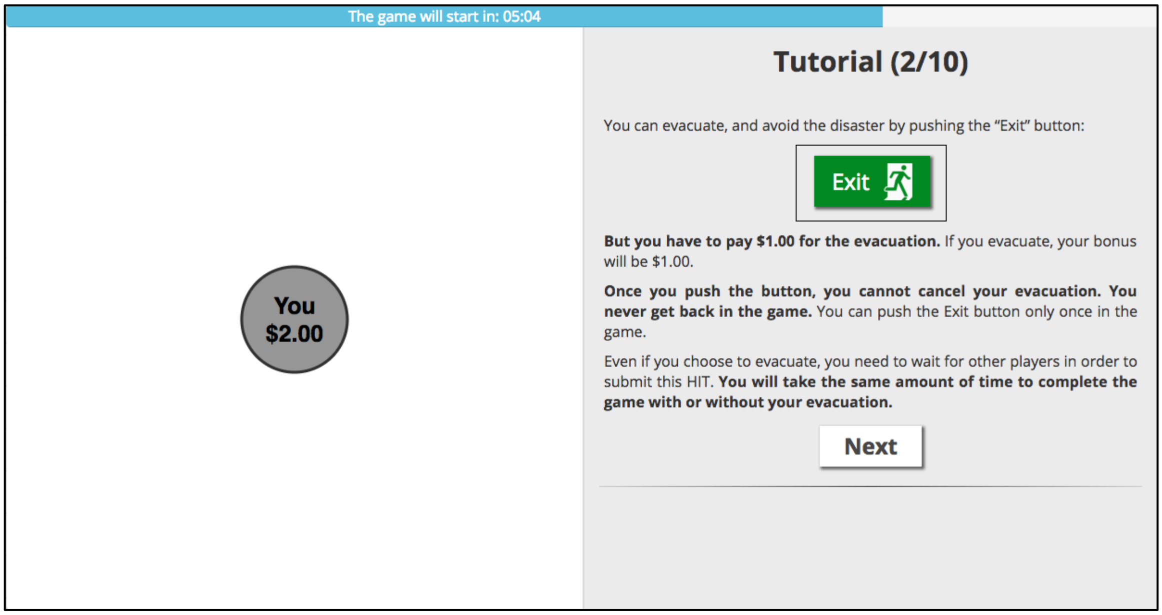

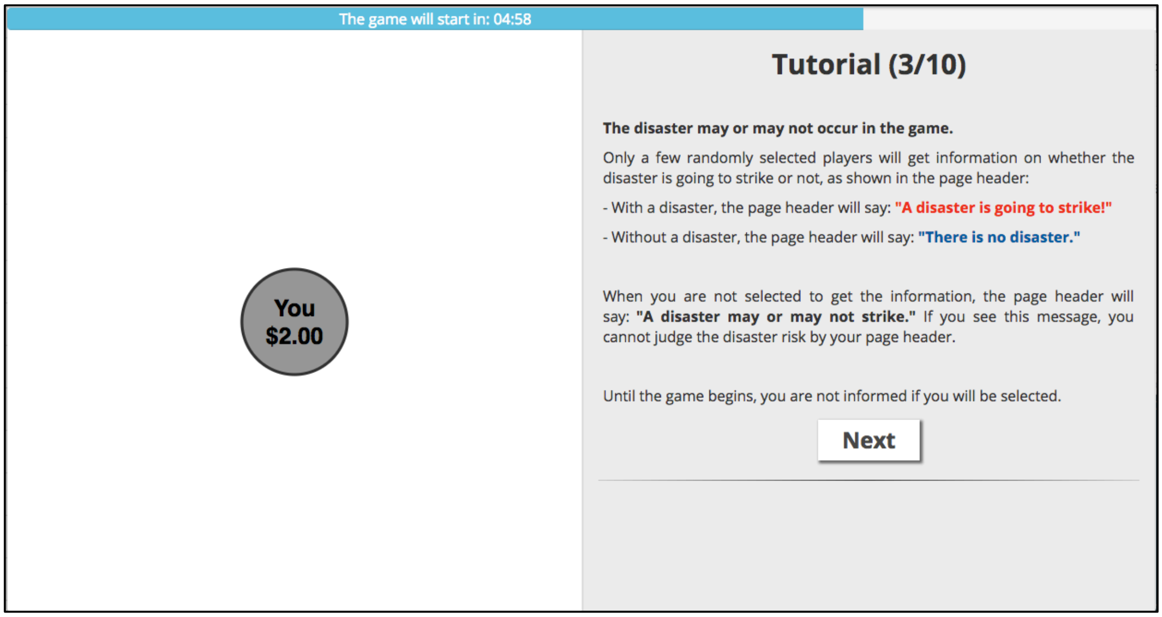
The game will start in: 04:58
You
$2.00
Tutorial (3/10)
The disaster may or may not occur in the game.
Only a few randomly selected players will get information on whether the disaster is going to strike or not, as shown in the page header:
- With a disaster, the page header will say: "A disaster is going to strike!"
- Without a disaster, the page header will say: "There is no disaster."
When you are not selected to get the information, the page header will say: "A disaster may or may not strike." If you see this message, you cannot judge the disaster risk by your page header.
Until the game begins, you are not informed if you will be selected.
Next

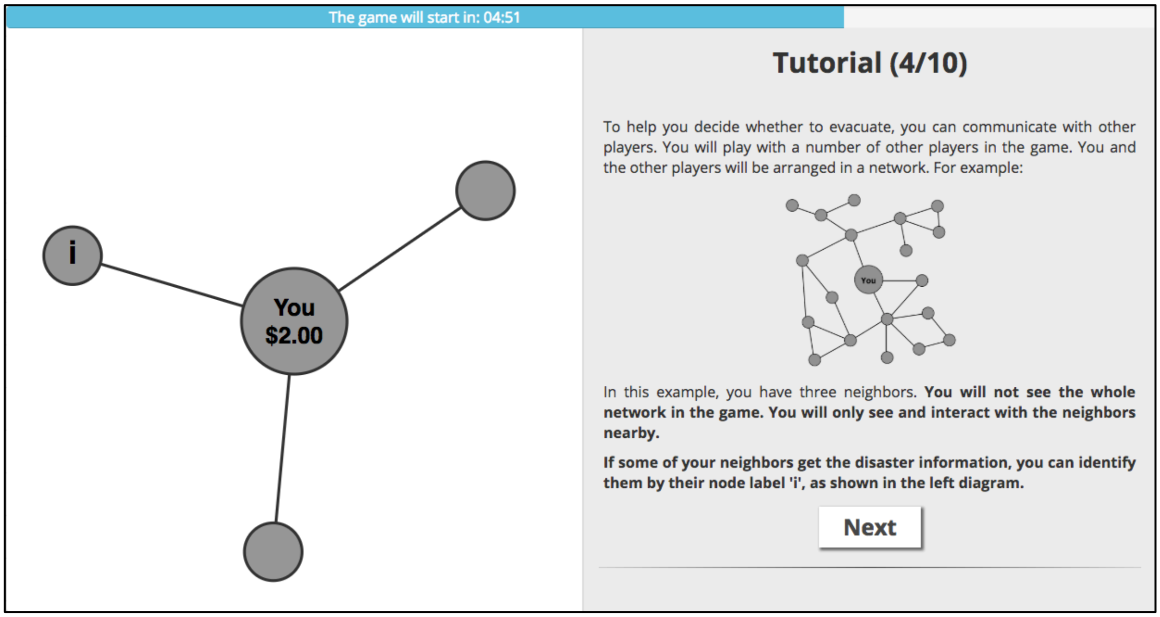
The game will start in: 04:51
i
You
$2.00
Tutorial (4/10)
To help you decide whether to evacuate, you can communicate with other players. You will play with a number of other players in the game. You and the other players will be arranged in a network. For example:
You
In this example, you have three neighbors. You will not see the whole network in the game. You will only see and interact with the neighbors nearby.
If some of your neighbors get the disaster information, you can identify them by their node label 'i', as shown in the left diagram.
Next

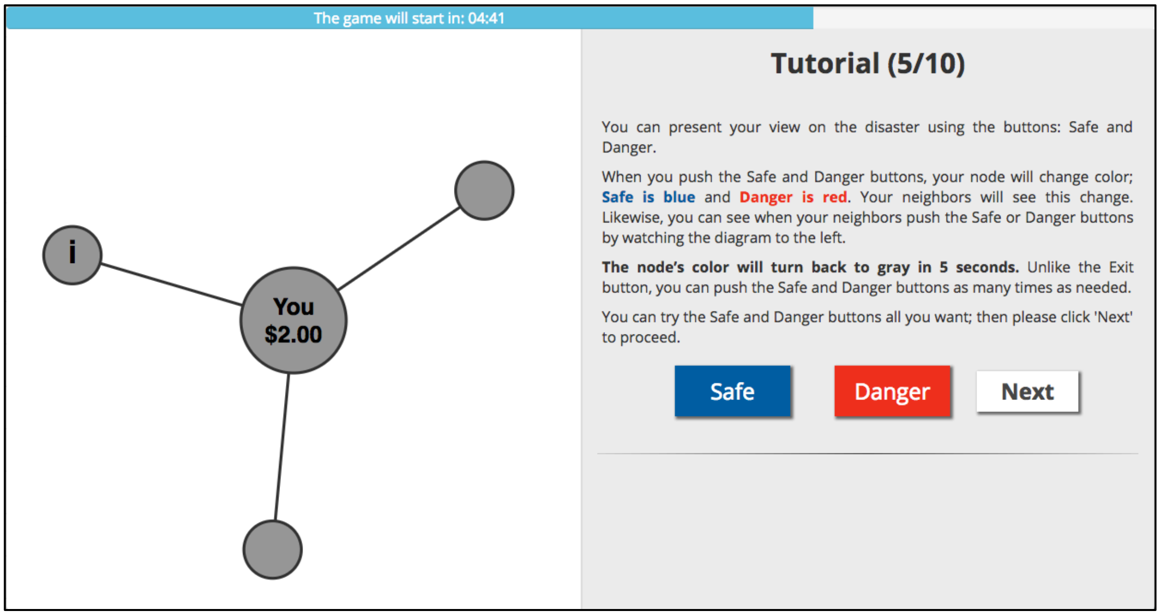
The game will start in: 04:41
i
You
$2.00
Tutorial (5/10)
You can present your view on the disaster using the buttons: Safe and Danger.
When you push the Safe and Danger buttons, your node will change color; Safe is blue and Danger is red. Your neighbors will see this change. Likewise, you can see when your neighbors push the Safe or Danger buttons by watching the diagram to the left.
The node's color will turn back to gray in 5 seconds. Unlike the Exit button, you can push the Safe and Danger buttons as many times as needed.
You can try the Safe and Danger buttons all you want; then please click 'Next' to proceed.
Safe
Danger
Next

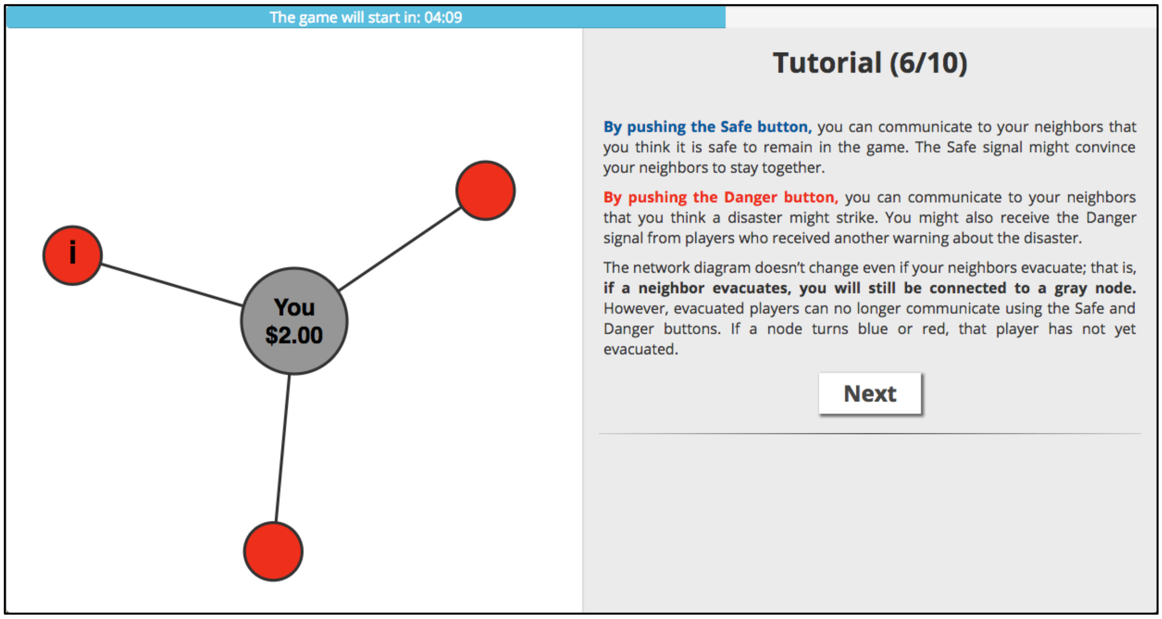
The game will start in: 04:09
i
You
$2.00
Tutorial (6/10)
By pushing the Safe button, you can communicate to your neighbors that you think it is safe to remain in the game. The Safe signal might convince your neighbors to stay together.
By pushing the Danger button, you can communicate to your neighbors that you think a disaster might strike. You might also receive the Danger signal from players who received another warning about the disaster.
The network diagram doesn't change even if your neighbors evacuate; that is, if a neighbor evacuates, you will still be connected to a gray node. However, evacuated players can no longer communicate using the Safe and Danger buttons. If a node turns blue or red, that player has not yet evacuated.
Next

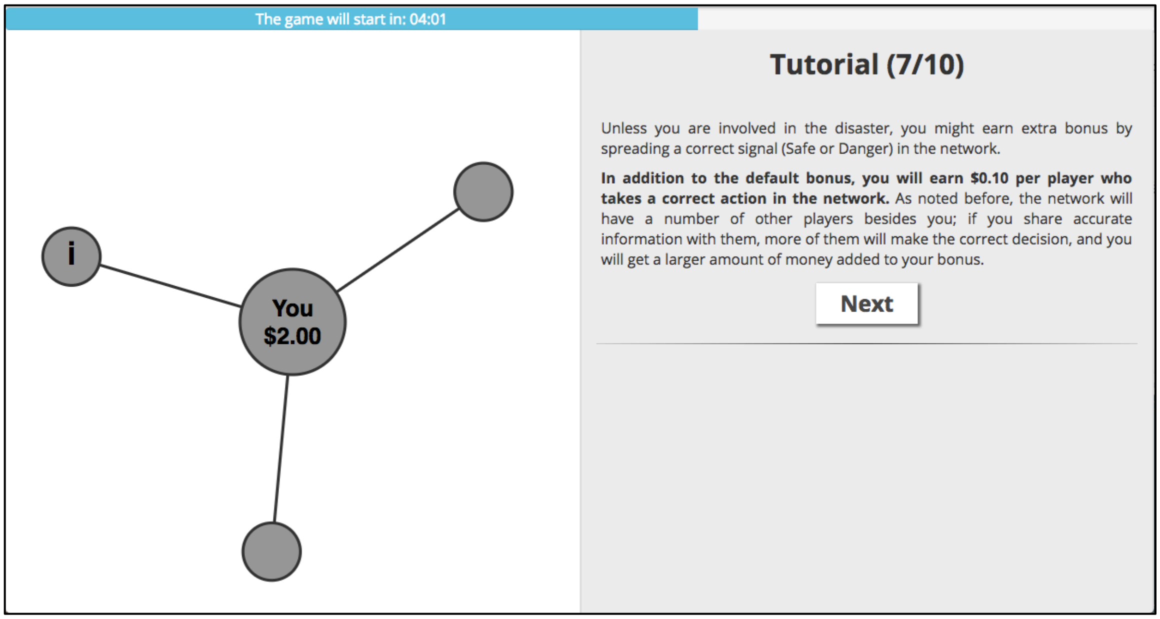
The game will start in: 04:01
i
You
$2.00
Tutorial (7/10)
Unless you are involved in the disaster, you might earn extra bonus by spreading a correct signal (Safe or Danger) in the network.
In addition to the default bonus, you will earn $0.10 per player who takes a correct action in the network. As noted before, the network will have a number of other players besides you; if you share accurate information with them, more of them will make the correct decision, and you will get a larger amount of money added to your bonus.
Next

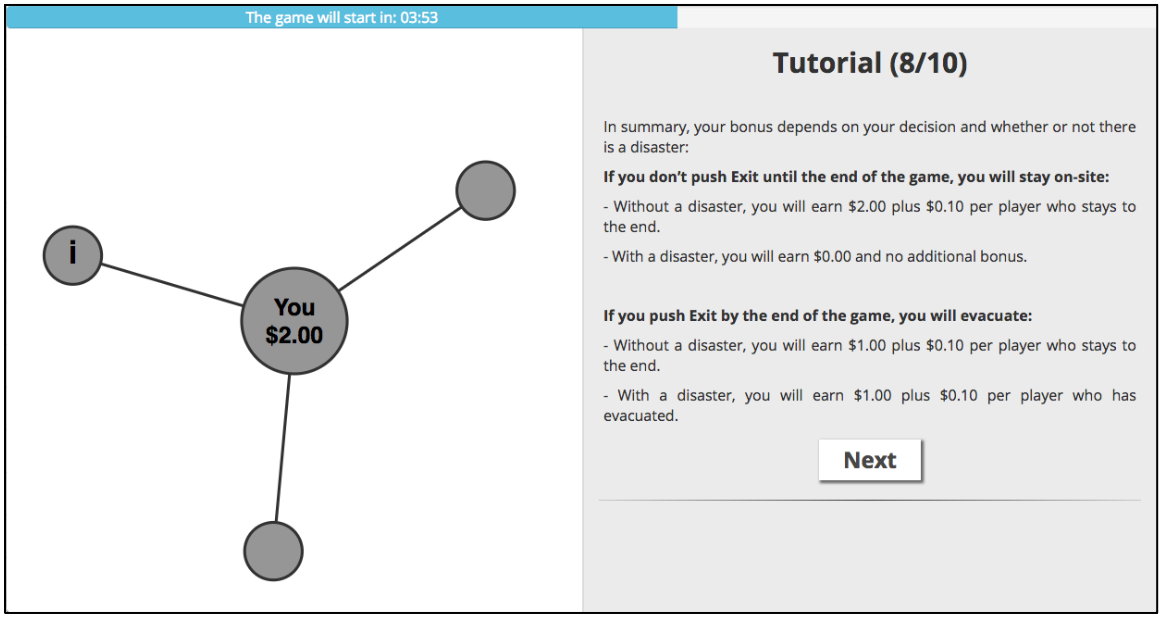
The game will start in: 03:53
i
You
$2.00
Tutorial (8/10)
In summary, your bonus depends on your decision and whether or not there is a disaster:
If you don't push Exit until the end of the game, you will stay on-site:
- Without a disaster, you will earn $2.00 plus $0.10 per player who stays to the end.
- With a disaster, you will earn $0.00 and no additional bonus.
If you push Exit by the end of the game, you will evacuate:
- Without a disaster, you will earn $1.00 plus $0.10 per player who stays to the end.
- With a disaster, you will earn $1.00 plus $0.10 per player who has evacuated.
Next

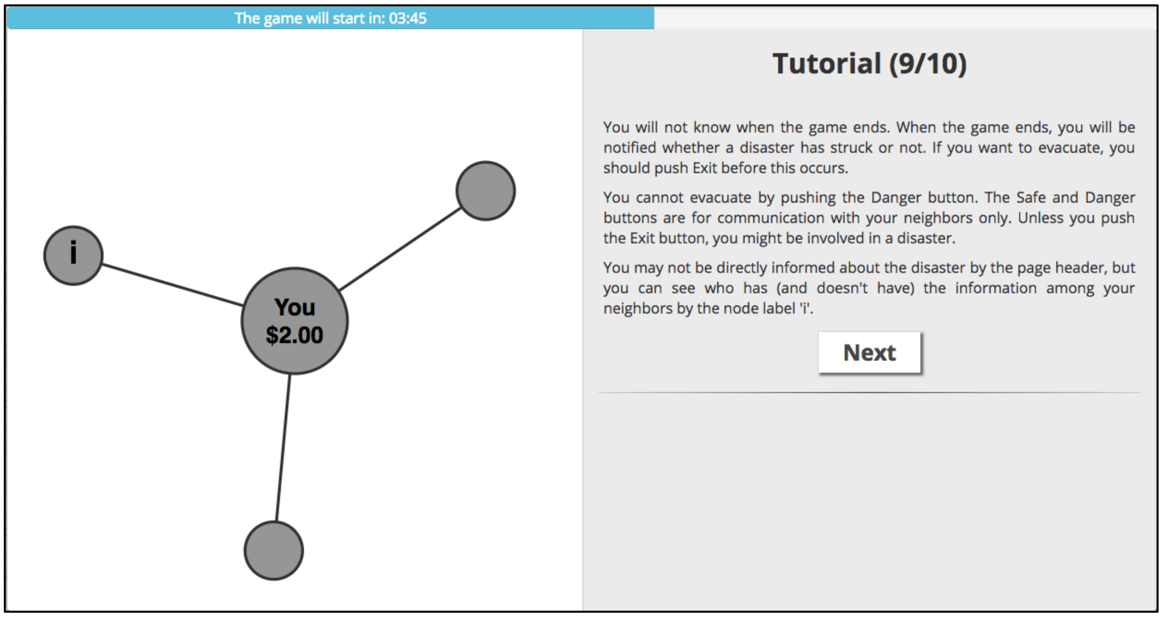
The game will start in: 03:45
Tutorial (9/10)
You will not know when the game ends. When the game ends, you will be notified whether a disaster has struck or not. If you want to evacuate, you should push Exit before this occurs.
You cannot evacuate by pushing the Danger button. The Safe and Danger buttons are for communication with your neighbors only. Unless you push the Exit button, you might be involved in a disaster.
You may not be directly informed about the disaster by the page header, but you can see who has (and doesn't have) the information among your neighbors by the node label 'i'.
Next
i
You
$2.00

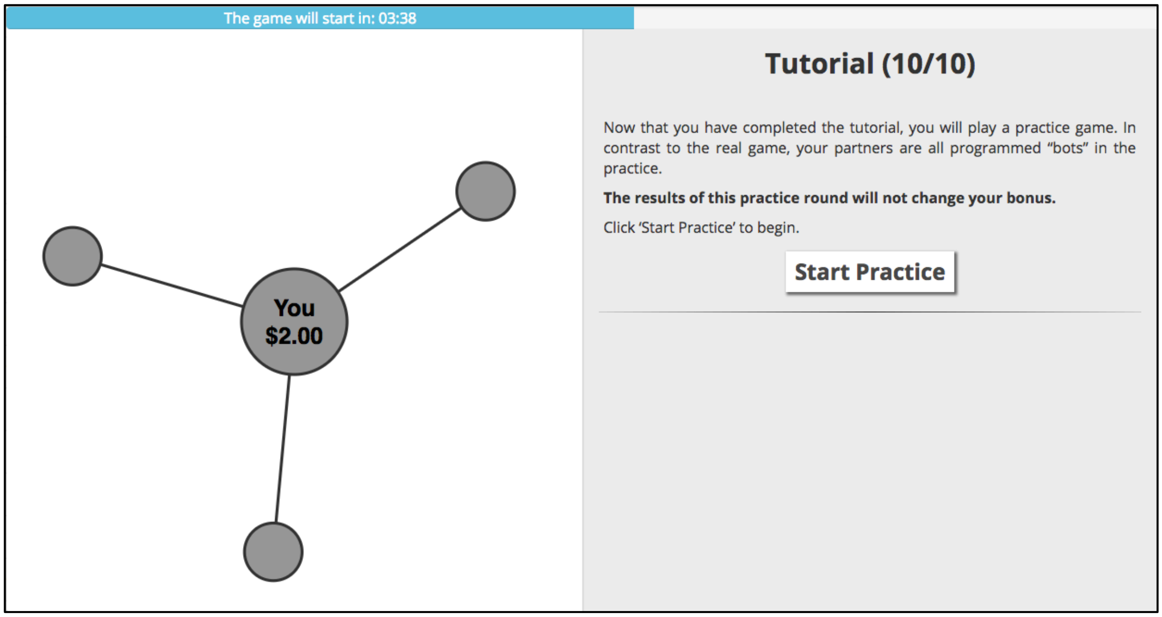
The game will start in: 03:38
Tutorial (10/10)
Now that you have completed the tutorial, you will play a practice game. In contrast to the real game, your partners are all programmed “bots” in the practice.
The results of this practice round will not change your bonus.
Click ‘Start Practice’ to begin.
Start Practice
You
$2.00

Practice game view of normal players

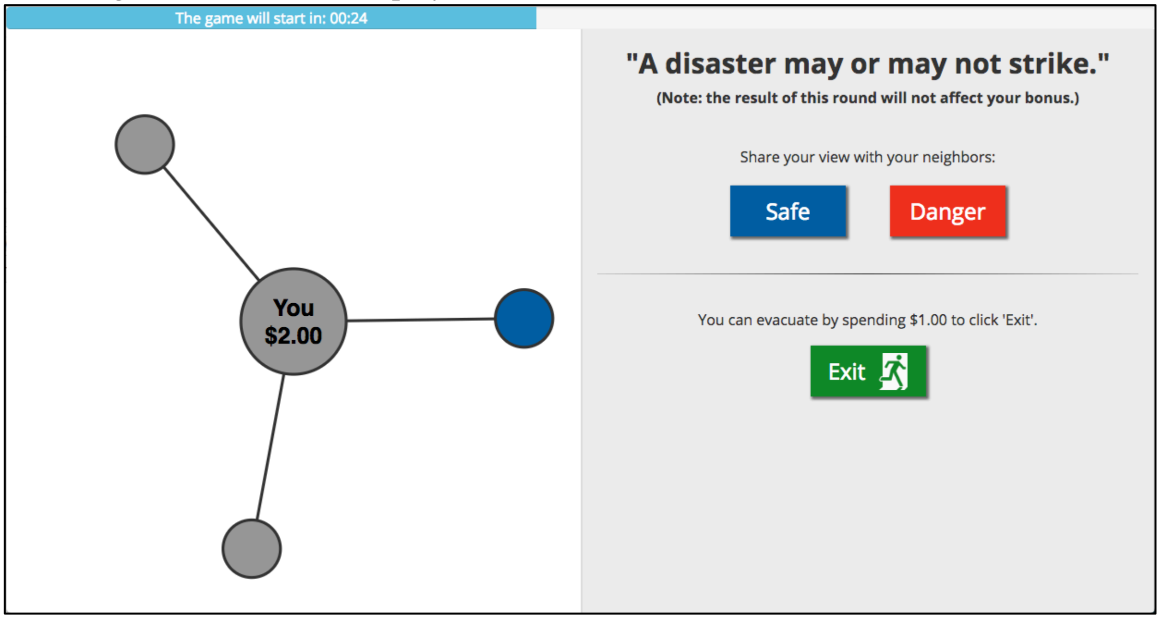

Practice game view of players chosen as informant

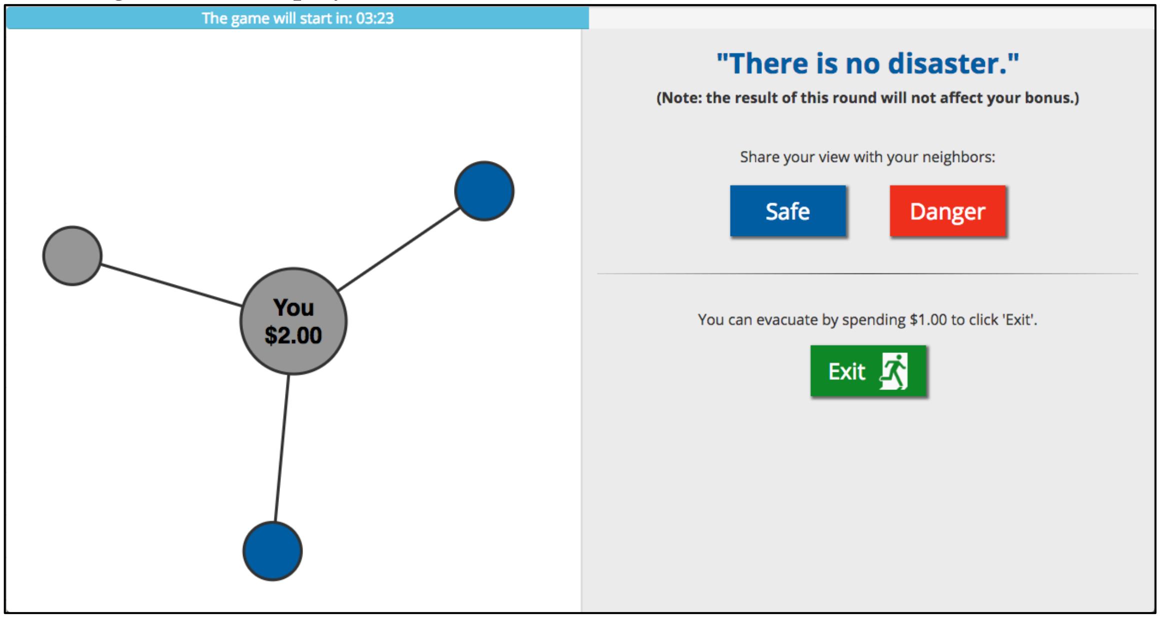

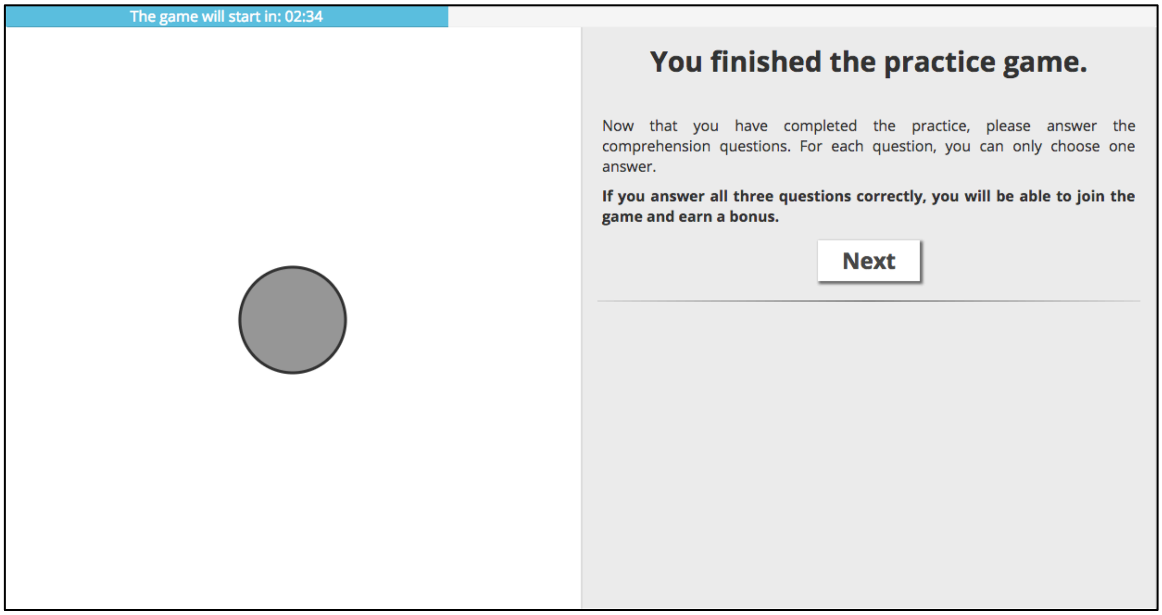
The game will start in: 02:34
You finished the practice game.
Now that you have completed the practice, please answer the comprehension questions. For each question, you can only choose one answer.
If you answer all three questions correctly, you will be able to join the game and earn a bonus.
Next

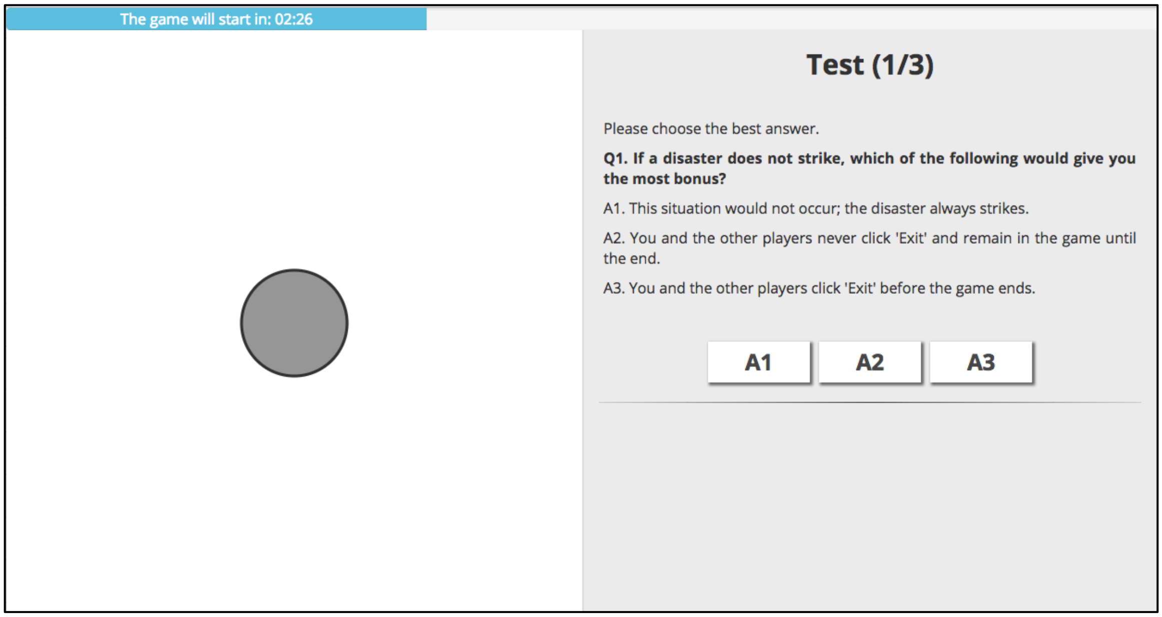
The game will start in: 02:26
Test (1/3)
Please choose the best answer.
Q1. If a disaster does not strike, which of the following would give you the most bonus?
A1. This situation would not occur; the disaster always strikes.
A2. You and the other players never click 'Exit' and remain in the game until the end.
A3. You and the other players click 'Exit' before the game ends.
A1
A2
A3

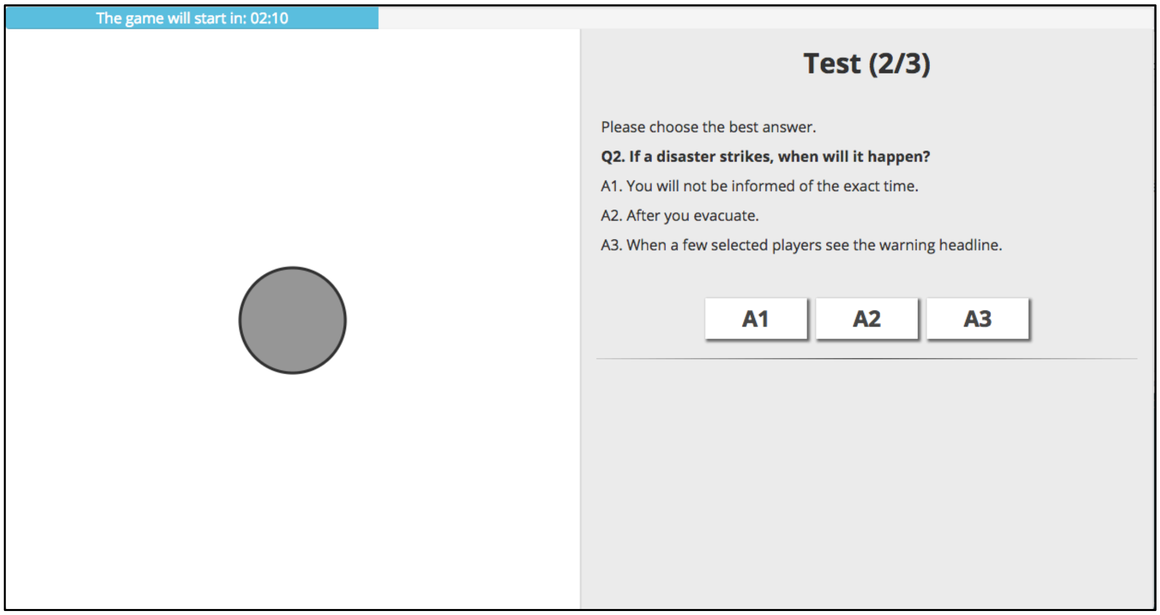
The game will start in: 02:10
Test (2/3)
Please choose the best answer.
Q2. If a disaster strikes, when will it happen?
A1. You will not be informed of the exact time.
A2. After you evacuate.
A3. When a few selected players see the warning headline.
A1
A2
A3

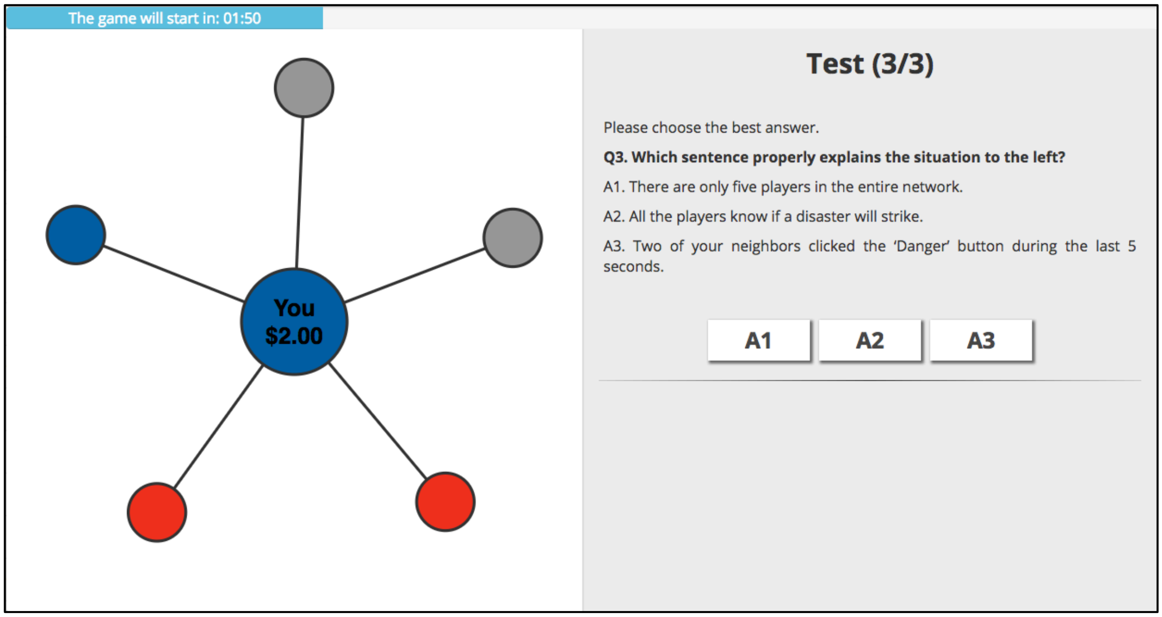
The game will start in: 01:50
You
$2.00
Test (3/3)
Please choose the best answer.
Q3. Which sentence properly explains the situation to the left?
A1. There are only five players in the entire network.
A2. All the players know if a disaster will strike.
A3. Two of your neighbors clicked the 'Danger' button during the last 5 seconds.
A1
A2
A3

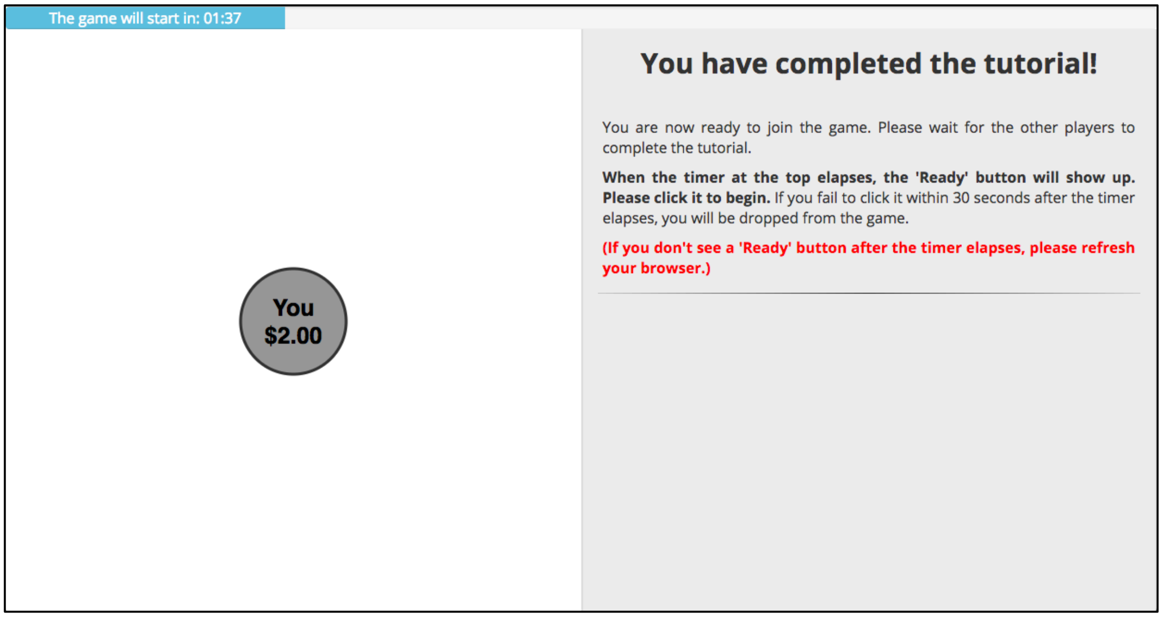

Game view of normal players

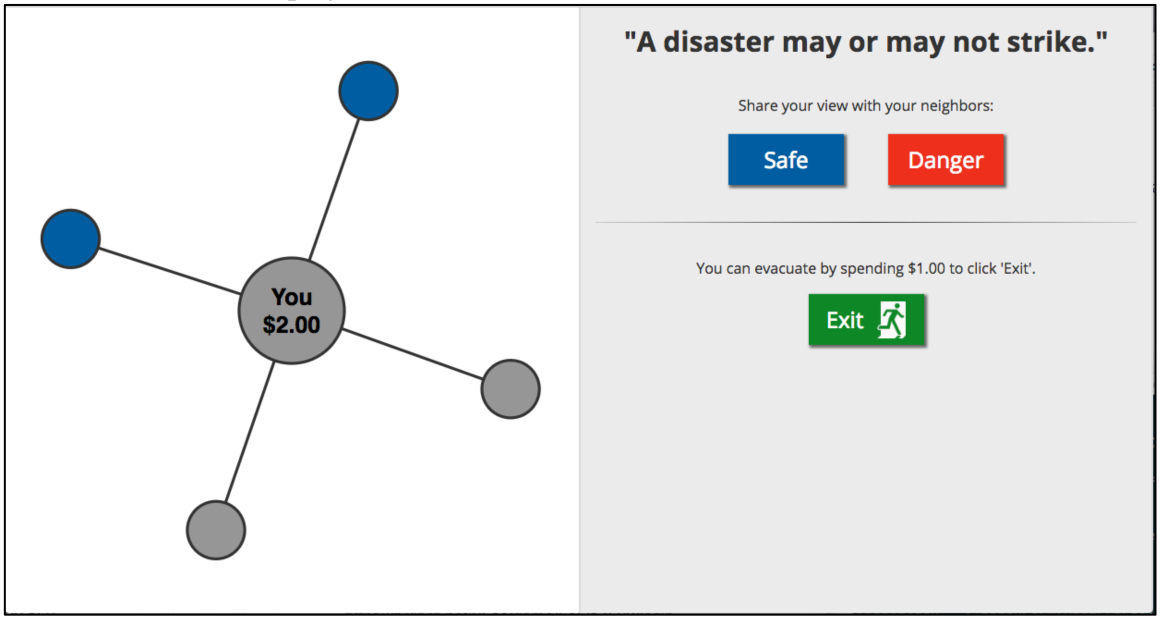

Game view of subjects chosen as informant

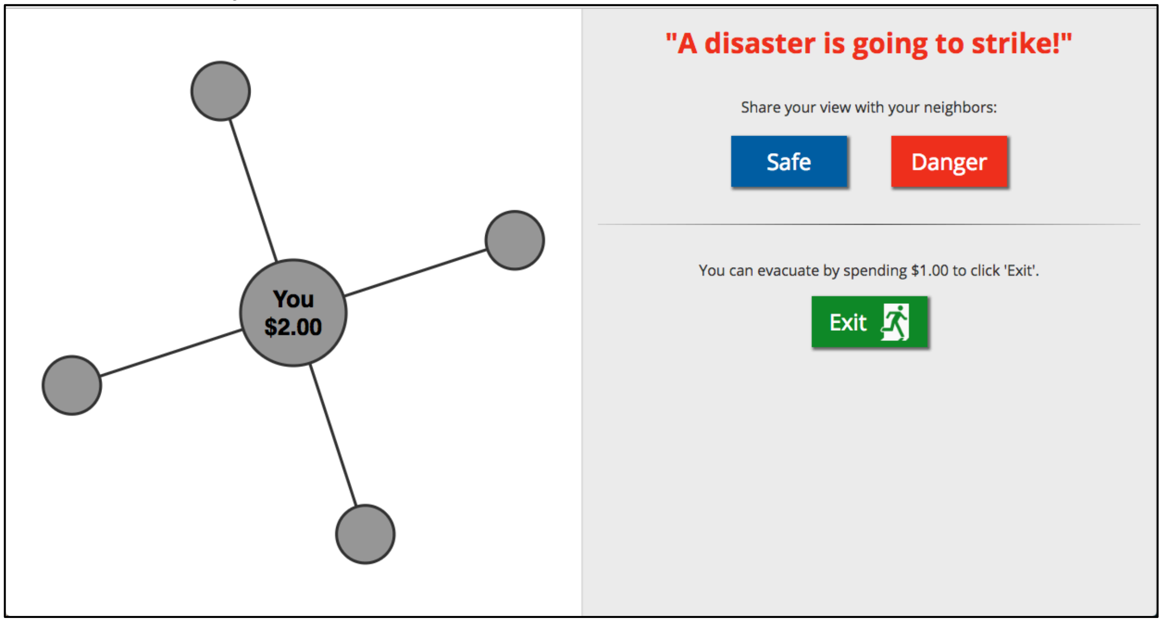

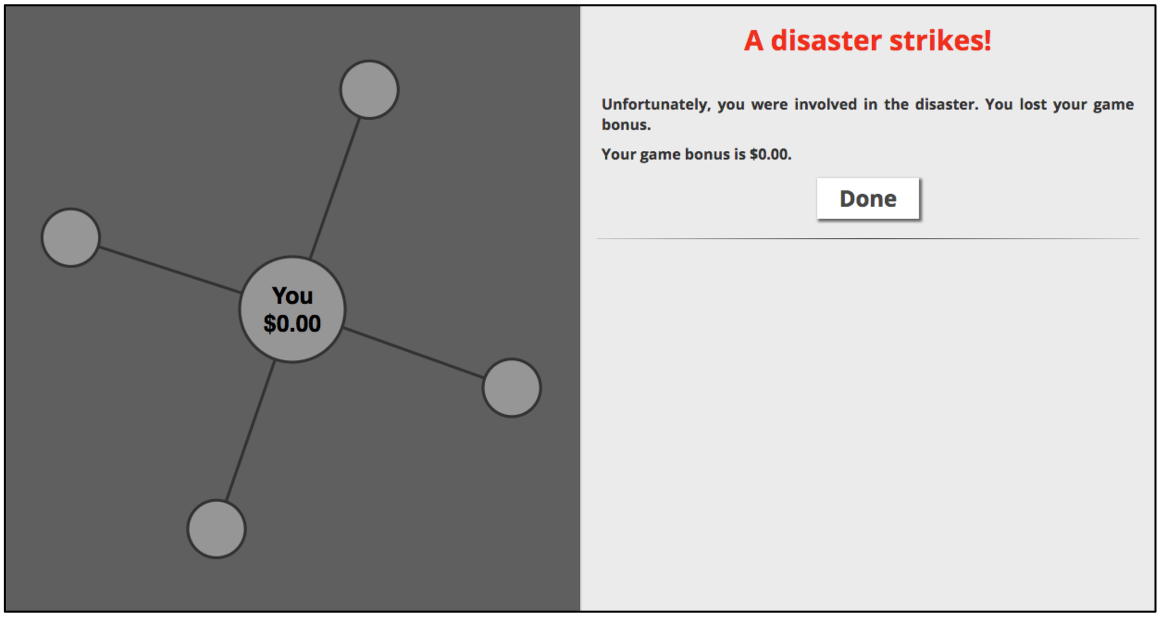

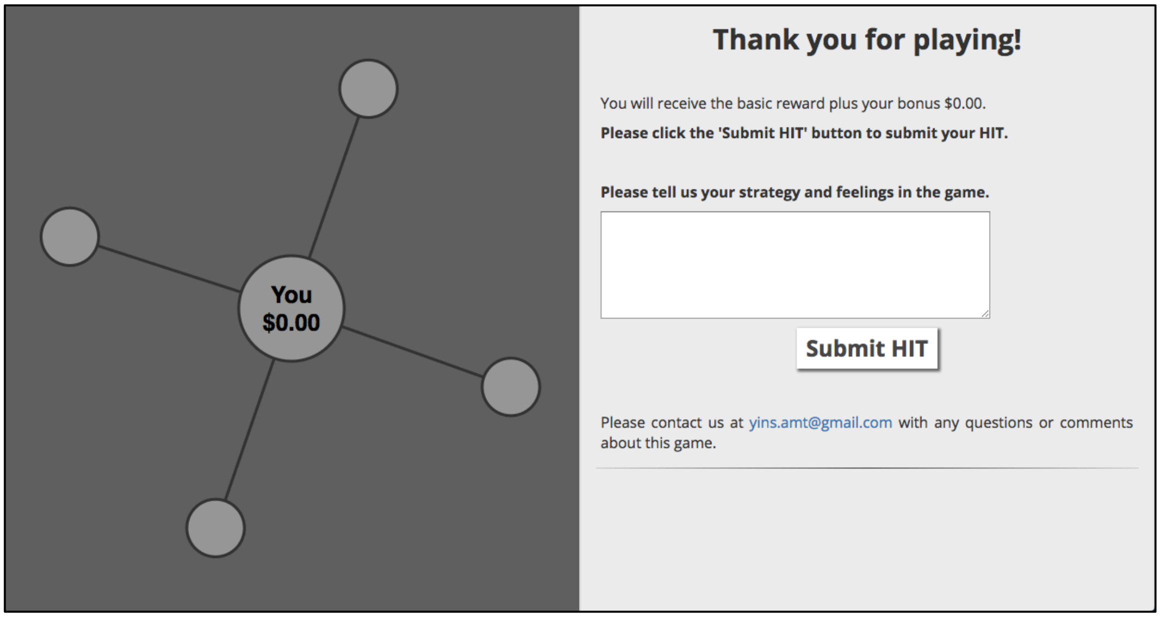

B. Independent condition

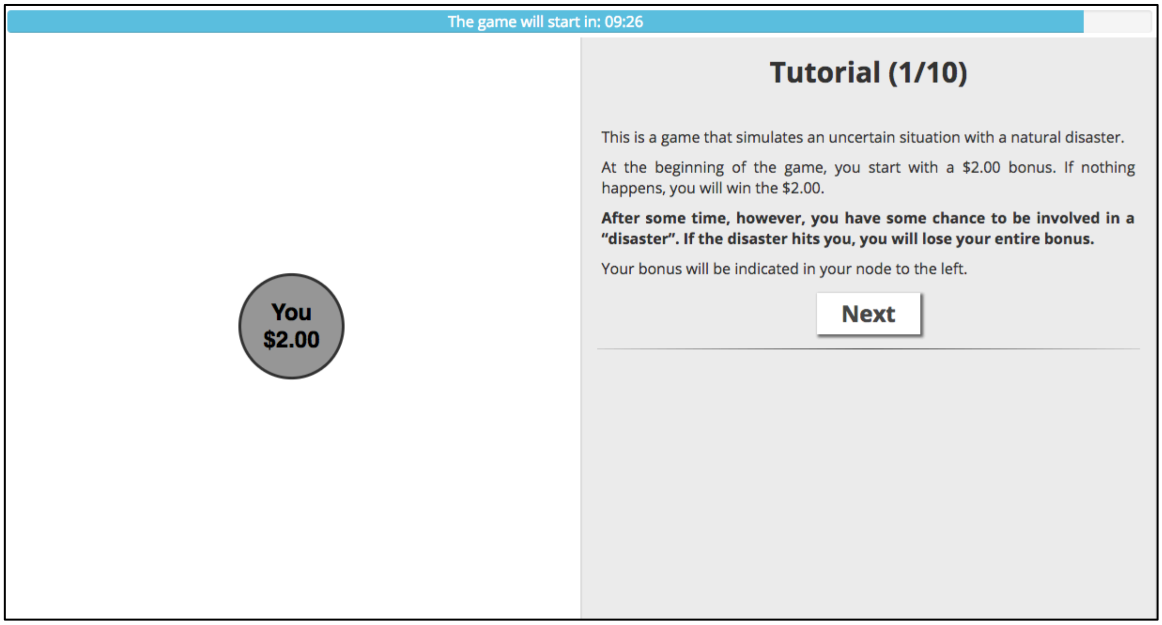

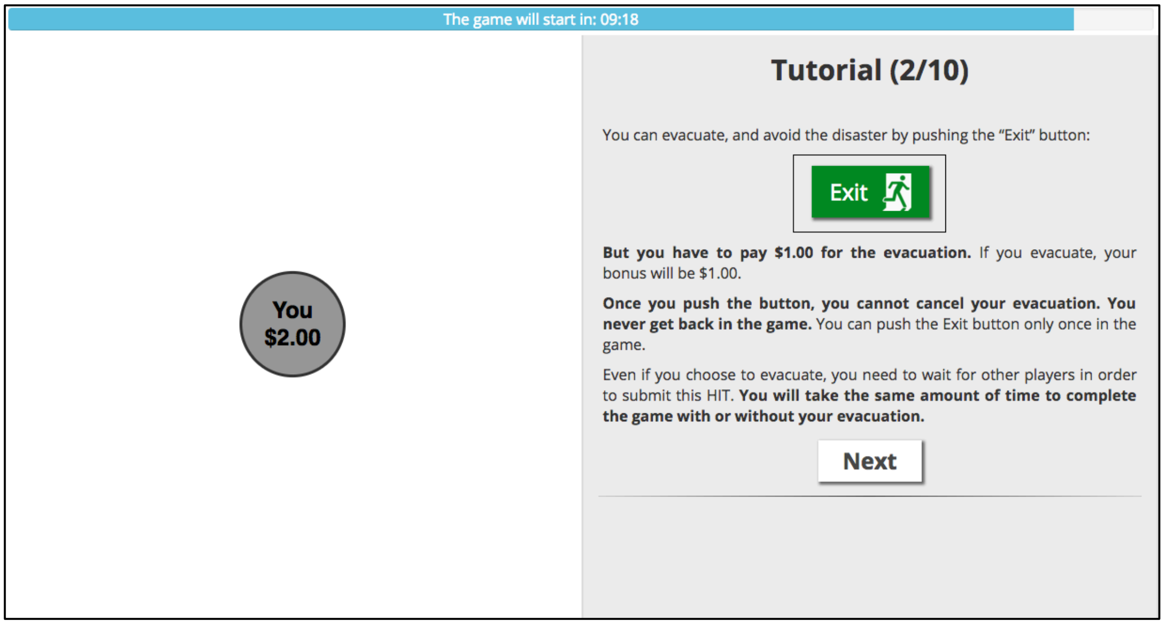
The game will start in: 09:18
You
$2.00
Tutorial (2/10)
You can evacuate, and avoid the disaster by pushing the "Exit" button:
Exit
But you have to pay $1.00 for the evacuation. If you evacuate, your bonus will be $1.00.
Once you push the button, you cannot cancel your evacuation. You never get back in the game. You can push the Exit button only once in the game.
Even if you choose to evacuate, you need to wait for other players in order to submit this HIT. You will take the same amount of time to complete the game with or without your evacuation.
Next

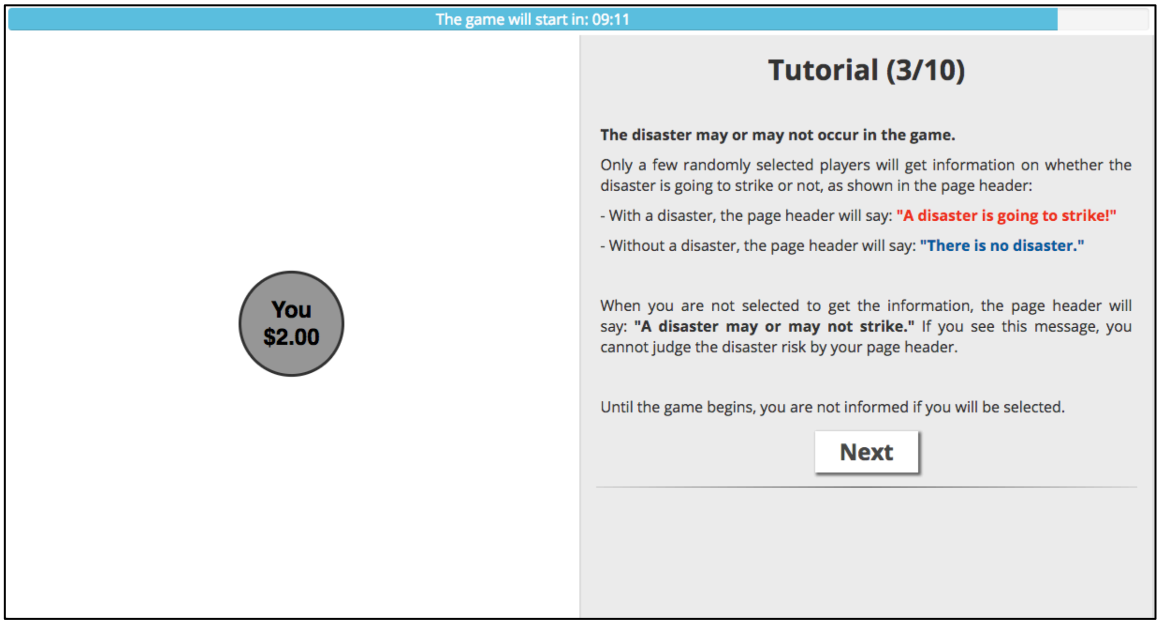
The game will start in: 09:11
You
$2.00
Tutorial (3/10)
The disaster may or may not occur in the game.
Only a few randomly selected players will get information on whether the disaster is going to strike or not, as shown in the page header:
- With a disaster, the page header will say: "A disaster is going to strike!"
- Without a disaster, the page header will say: "There is no disaster."
When you are not selected to get the information, the page header will say: "A disaster may or may not strike." If you see this message, you cannot judge the disaster risk by your page header.
Until the game begins, you are not informed if you will be selected.
Next

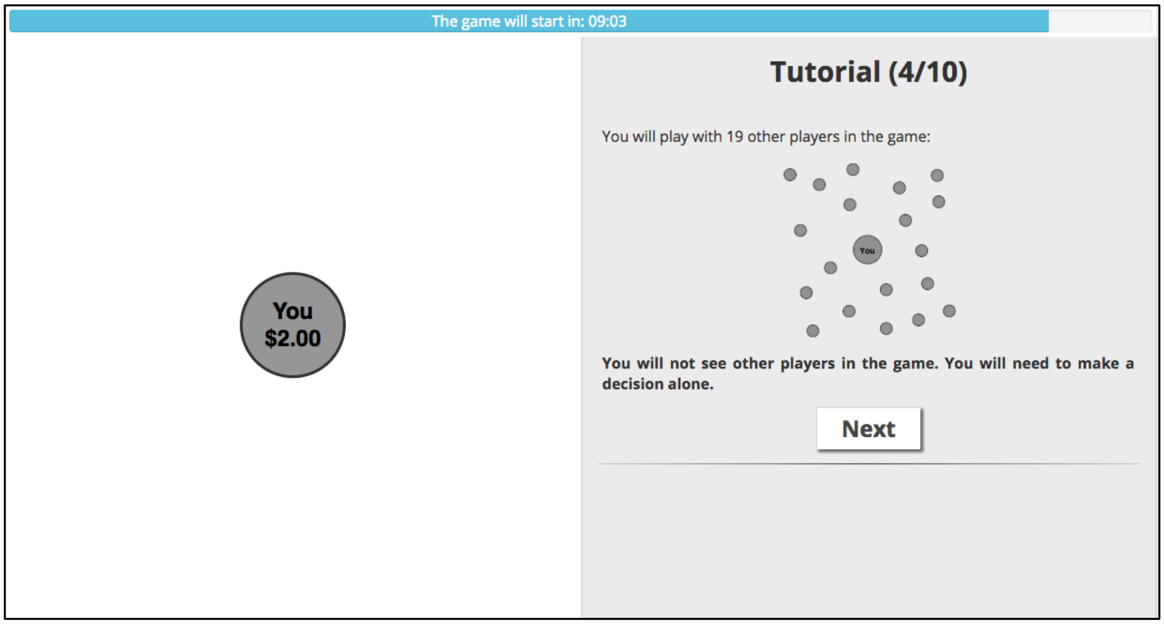
The game will start in: 09:03
You
$2.00
Tutorial (4/10)
You will play with 19 other players in the game:
You
You will not see other players in the game. You will need to make a decision alone.
Next

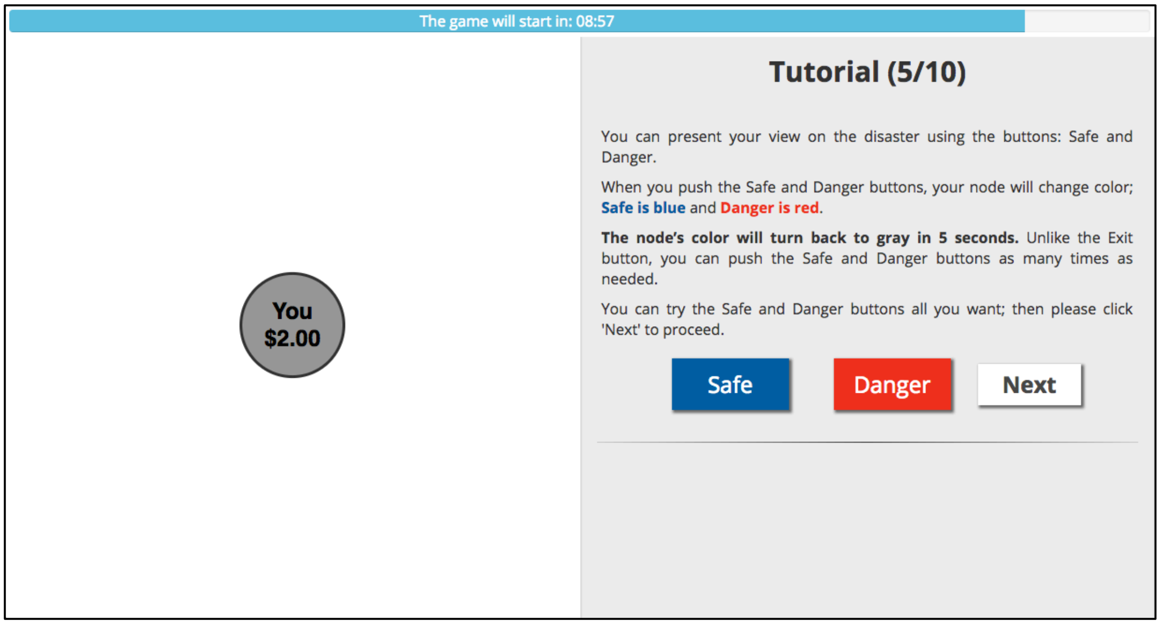
The game will start in: 08:57
You
$2.00
Tutorial (5/10)
You can present your view on the disaster using the buttons: Safe and Danger.
When you push the Safe and Danger buttons, your node will change color; Safe is blue and Danger is red.
The node's color will turn back to gray in 5 seconds. Unlike the Exit button, you can push the Safe and Danger buttons as many times as needed.
You can try the Safe and Danger buttons all you want; then please click 'Next' to proceed.
Safe
Danger
Next

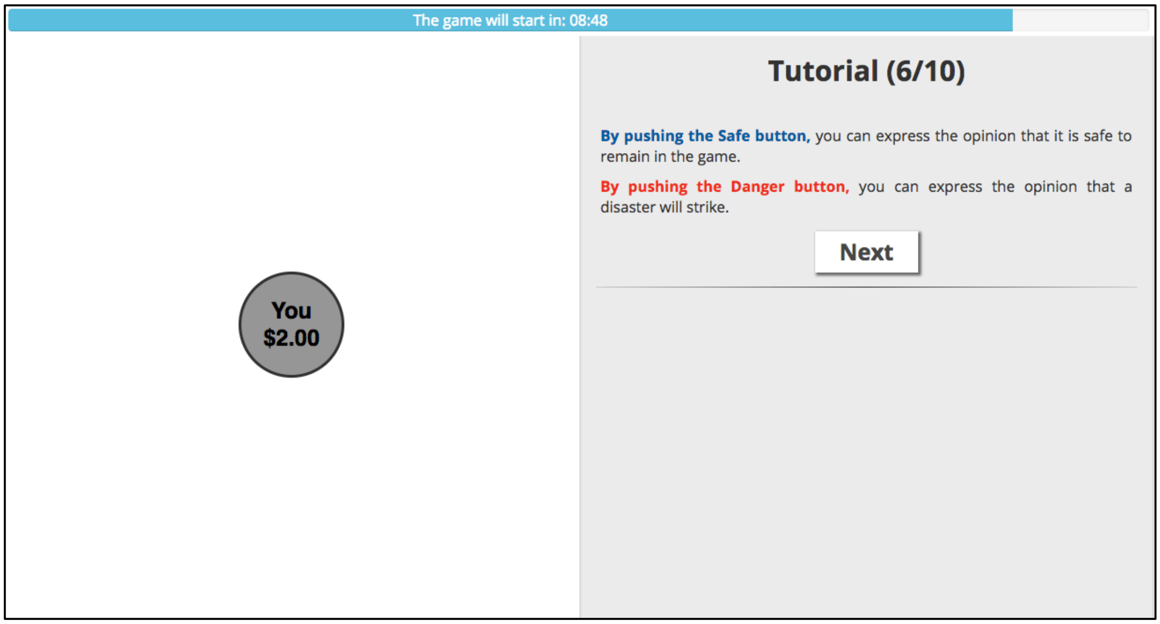
The game will start in: 08:48
Tutorial (6/10)
By pushing the Safe button, you can express the opinion that it is safe to remain in the game.
By pushing the Danger button, you can express the opinion that a disaster will strike.
Next
You
$2.00

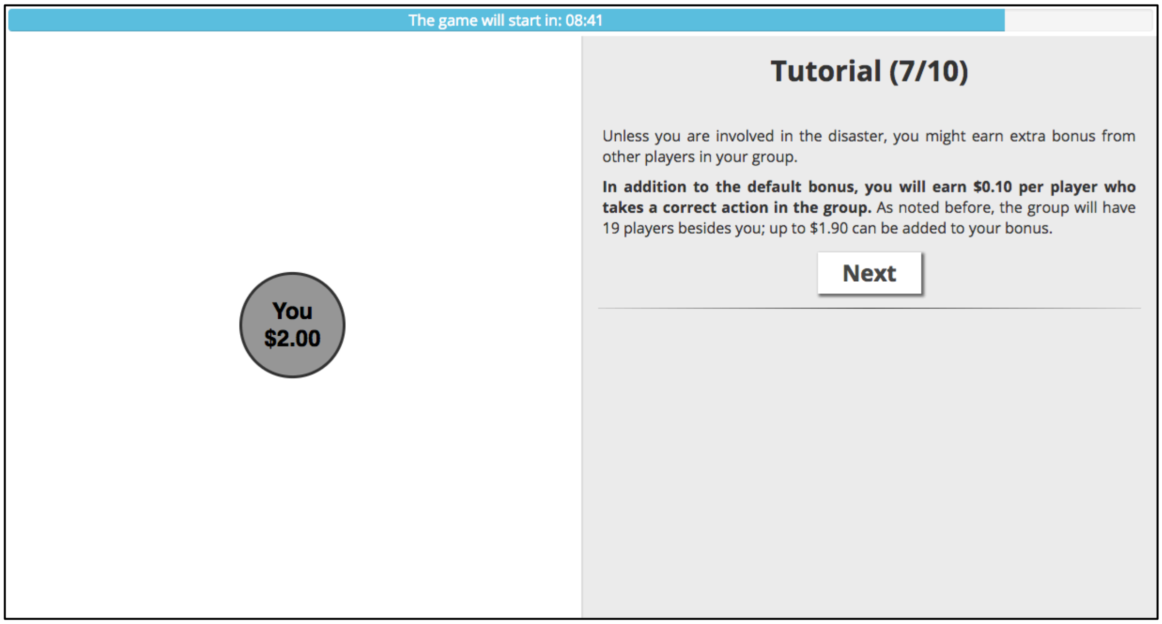
The game will start in: 08:41
Tutorial (7/10)
Unless you are involved in the disaster, you might earn extra bonus from other players in your group.
In addition to the default bonus, you will earn $0.10 per player who takes a correct action in the group. As noted before, the group will have 19 players besides you; up to $1.90 can be added to your bonus.
Next
You
$2.00

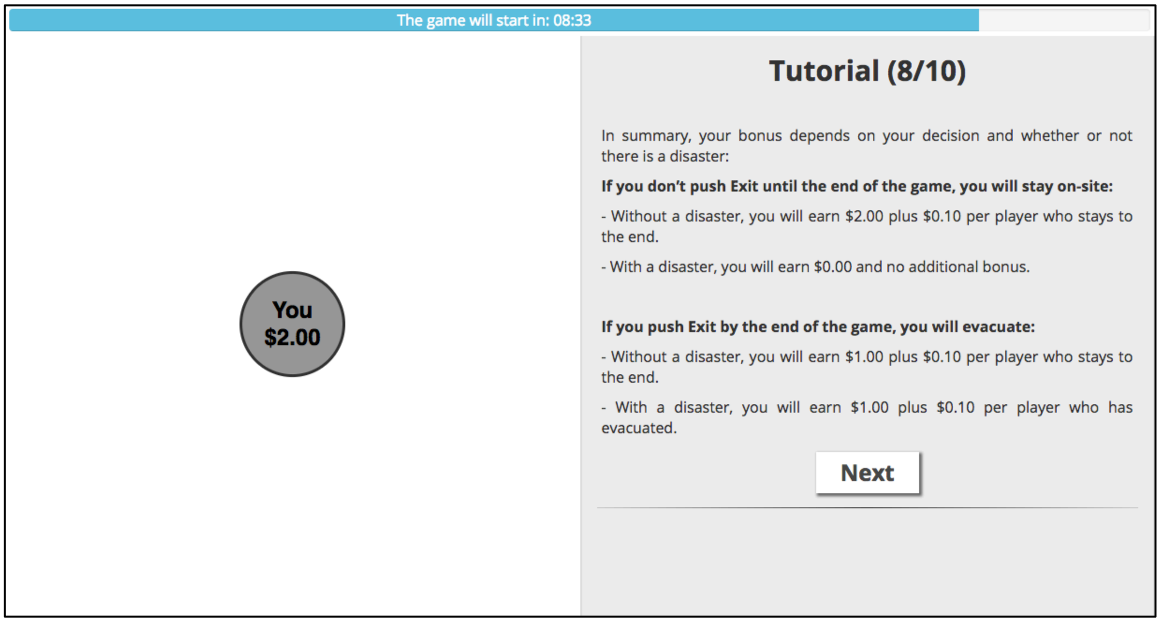
The game will start in: 08:33
You
$2.00
Tutorial (8/10)
In summary, your bonus depends on your decision and whether or not there is a disaster:
If you don't push Exit until the end of the game, you will stay on-site:
- Without a disaster, you will earn $2.00 plus $0.10 per player who stays to the end.
- With a disaster, you will earn $0.00 and no additional bonus.
If you push Exit by the end of the game, you will evacuate:
- Without a disaster, you will earn $1.00 plus $0.10 per player who stays to the end.
- With a disaster, you will earn $1.00 plus $0.10 per player who has evacuated.
Next

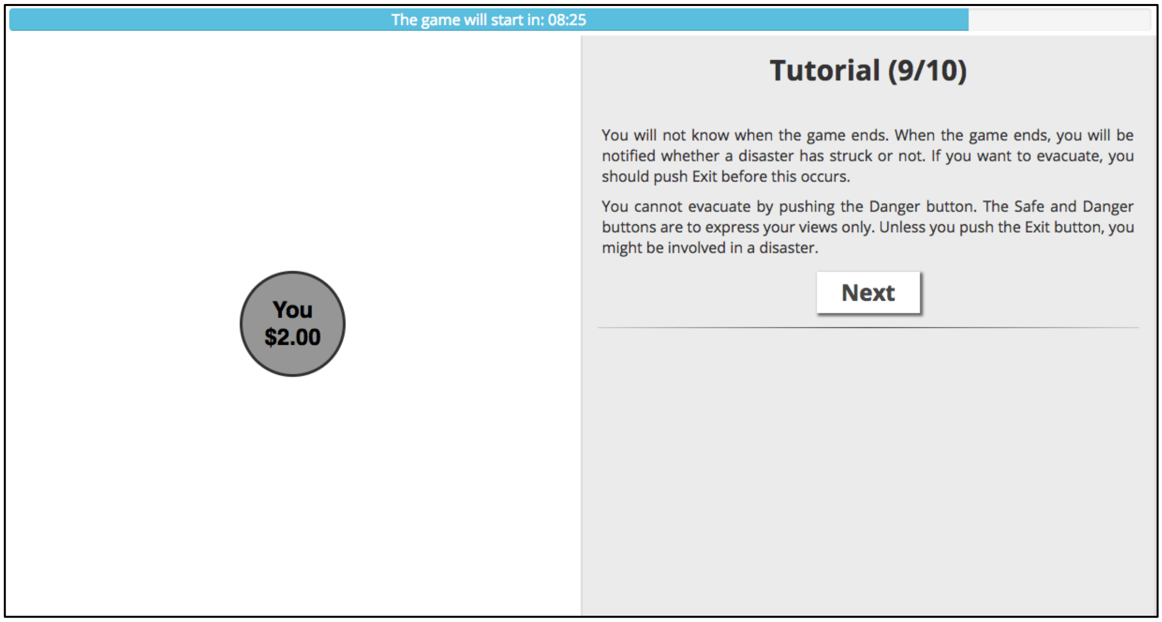
The game will start in: 08:25
You
$2.00
Tutorial (9/10)
You will not know when the game ends. When the game ends, you will be notified whether a disaster has struck or not. If you want to evacuate, you should push Exit before this occurs.
You cannot evacuate by pushing the Danger button. The Safe and Danger buttons are to express your views only. Unless you push the Exit button, you might be involved in a disaster.
Next

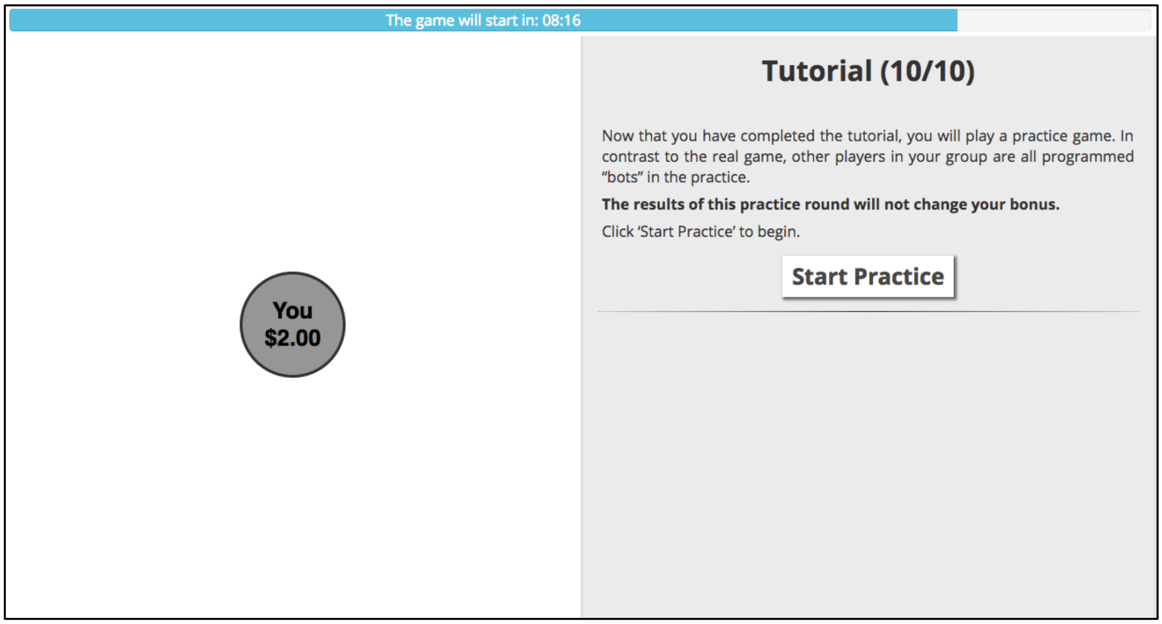
The game will start in: 08:16
Tutorial (10/10)
Now that you have completed the tutorial, you will play a practice game. In contrast to the real game, other players in your group are all programmed "bots" in the practice.
The results of this practice round will not change your bonus.
Click 'Start Practice' to begin.
Start Practice
You
$2.00

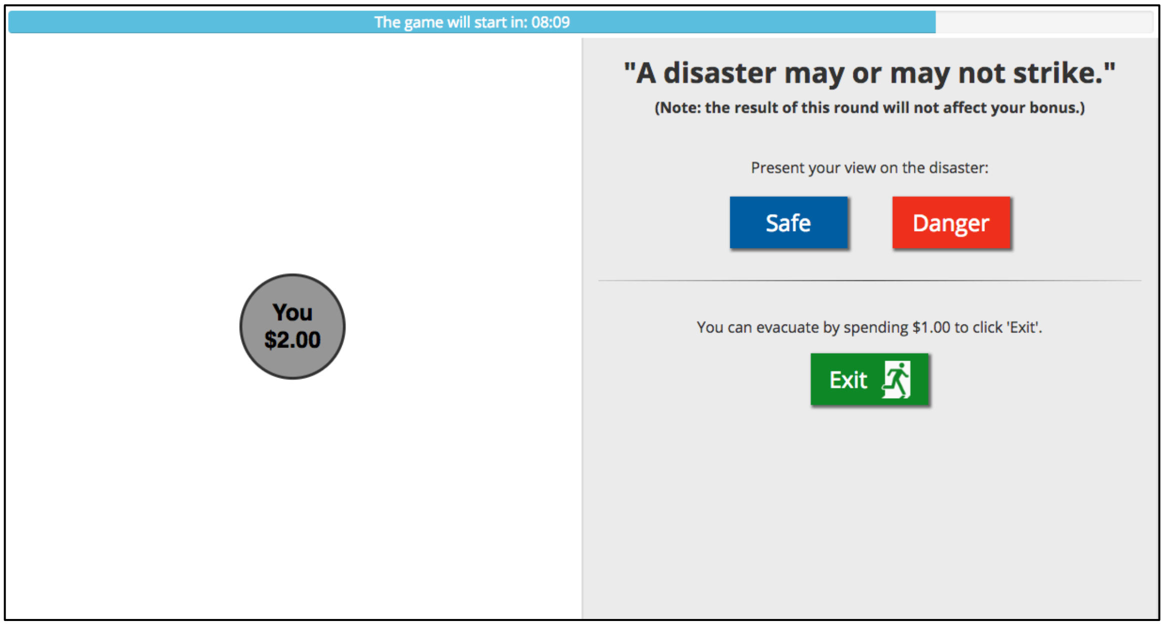
The game will start in: 08:09
"A disaster may or may not strike."
(Note: the result of this round will not affect your bonus.)
Present your view on the disaster:
Safe
Danger
You can evacuate by spending $1.00 to click 'Exit'.
Exit
You
$2.00

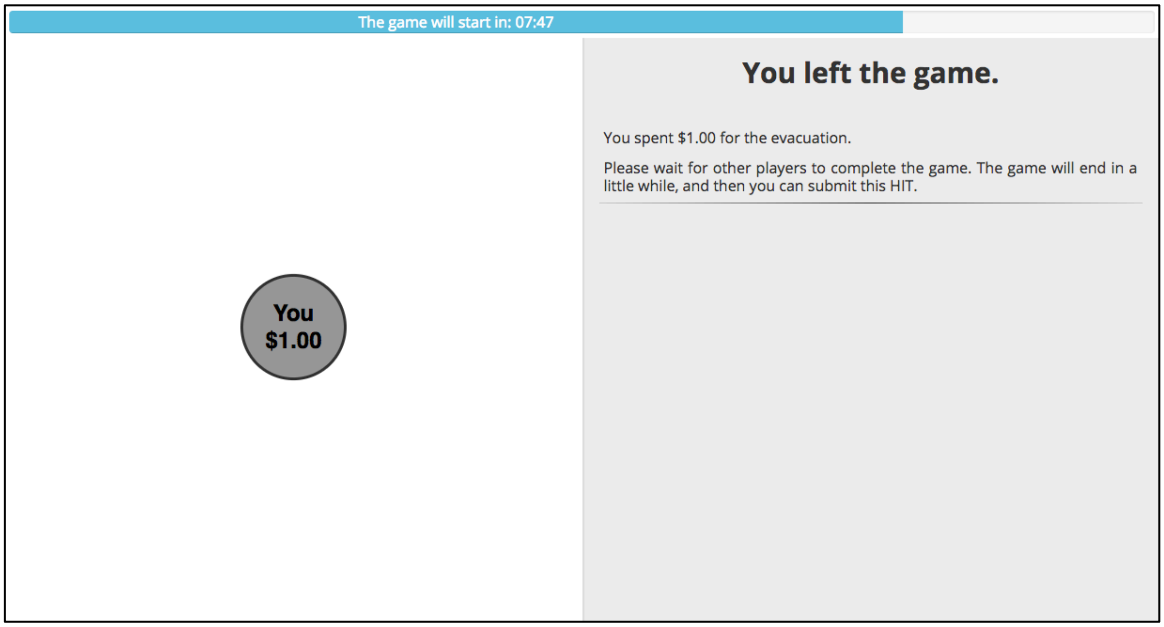
The game will start in: 07:47
You left the game.
You spent $1.00 for the evacuation.
Please wait for other players to complete the game. The game will end in a little while, and then you can submit this HIT.
You
$1.00

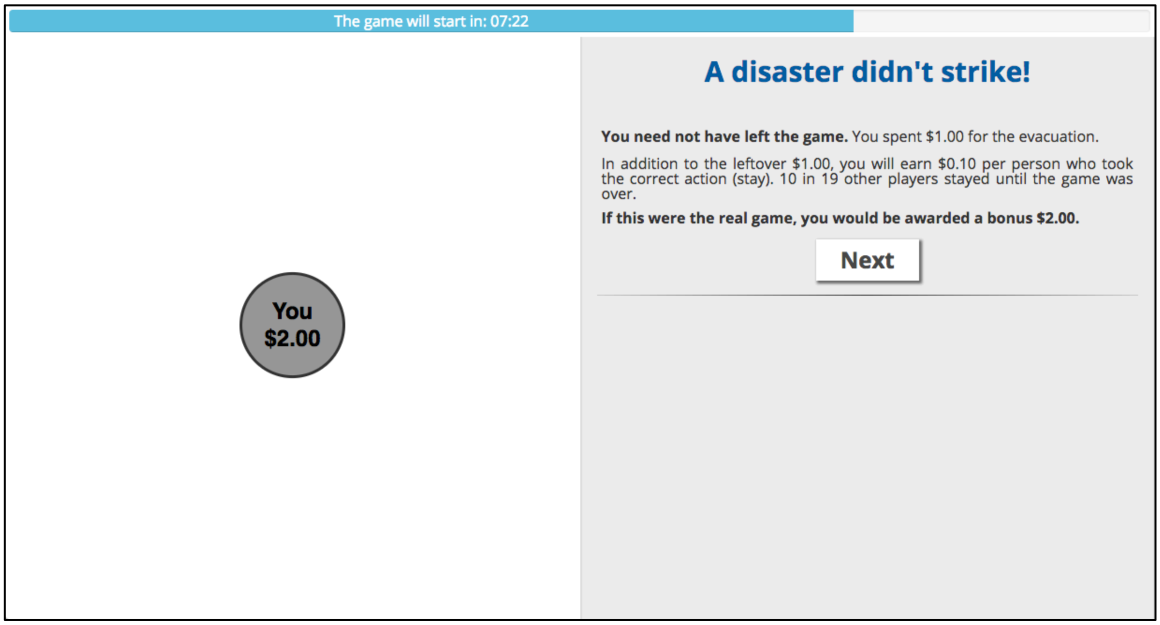
The game will start in: 07:22
A disaster didn't strike!
You need not have left the game. You spent $1.00 for the evacuation.
In addition to the leftover $1.00, you will earn $0.10 per person who took the correct action (stay). 10 in 19 other players stayed until the game was over.
If this were the real game, you would be awarded a bonus $2.00.
Next
You
$2.00

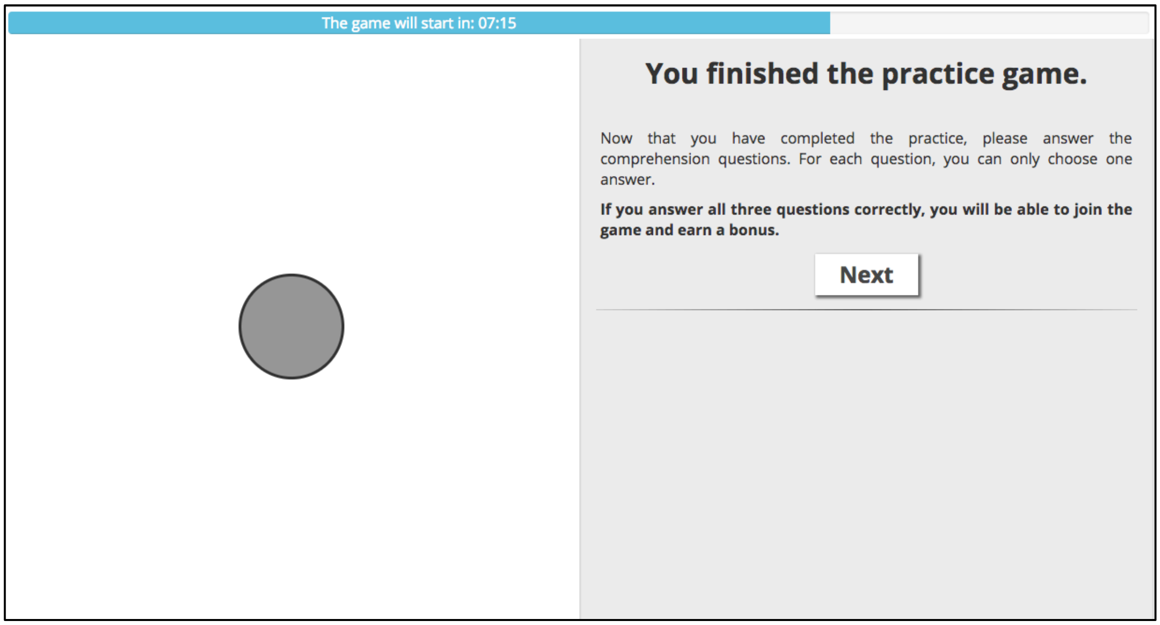
The game will start in: 07:15
You finished the practice game.
Now that you have completed the practice, please answer the comprehension questions. For each question, you can only choose one answer.
If you answer all three questions correctly, you will be able to join the game and earn a bonus.
Next

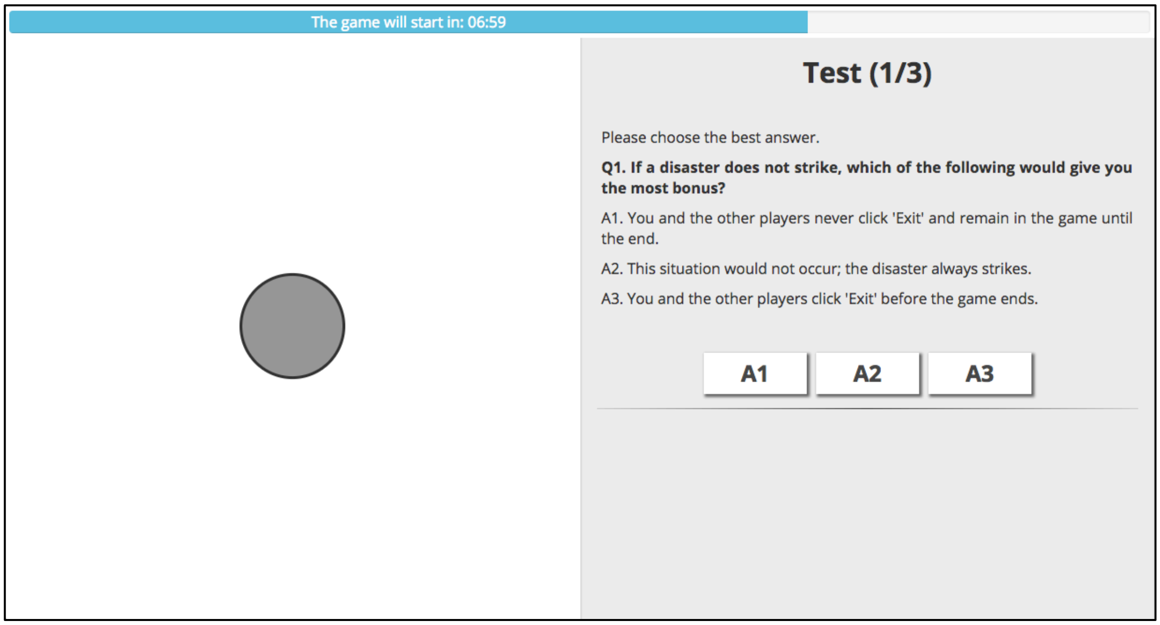
The game will start in: 06:59
Test (1/3)
Please choose the best answer.
Q1. If a disaster does not strike, which of the following would give you the most bonus?
A1. You and the other players never click 'Exit' and remain in the game until the end.
A2. This situation would not occur; the disaster always strikes.
A3. You and the other players click 'Exit' before the game ends.
A1
A2
A3

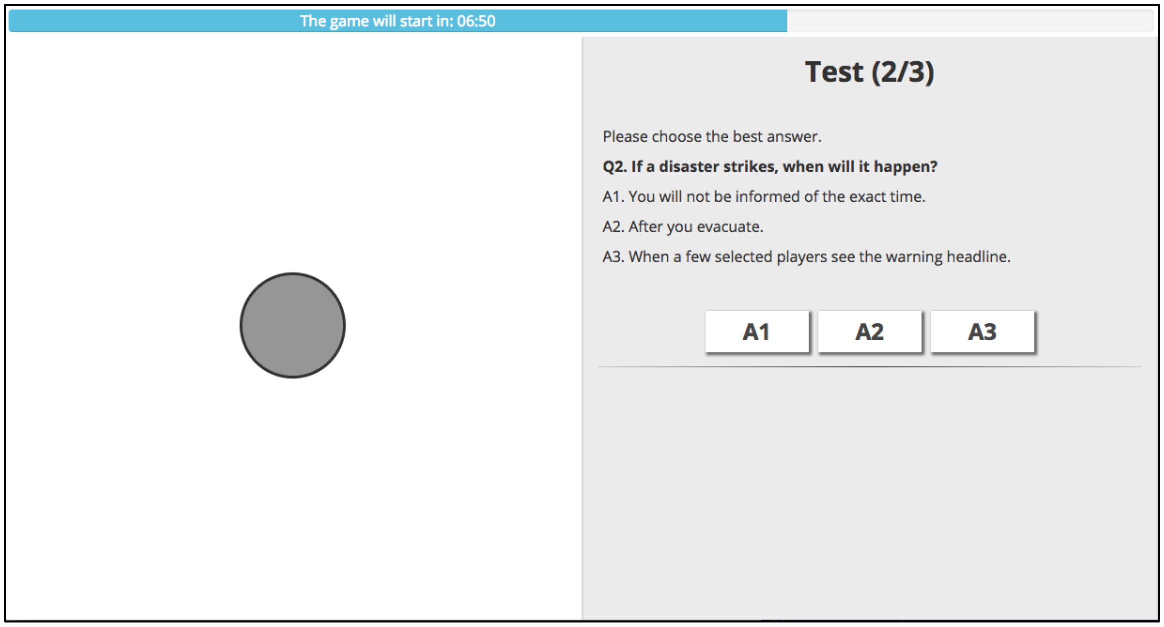
The game will start in: 06:50
Test (2/3)
Please choose the best answer.
Q2. If a disaster strikes, when will it happen?
A1. You will not be informed of the exact time.
A2. After you evacuate.
A3. When a few selected players see the warning headline.
A1
A2
A3

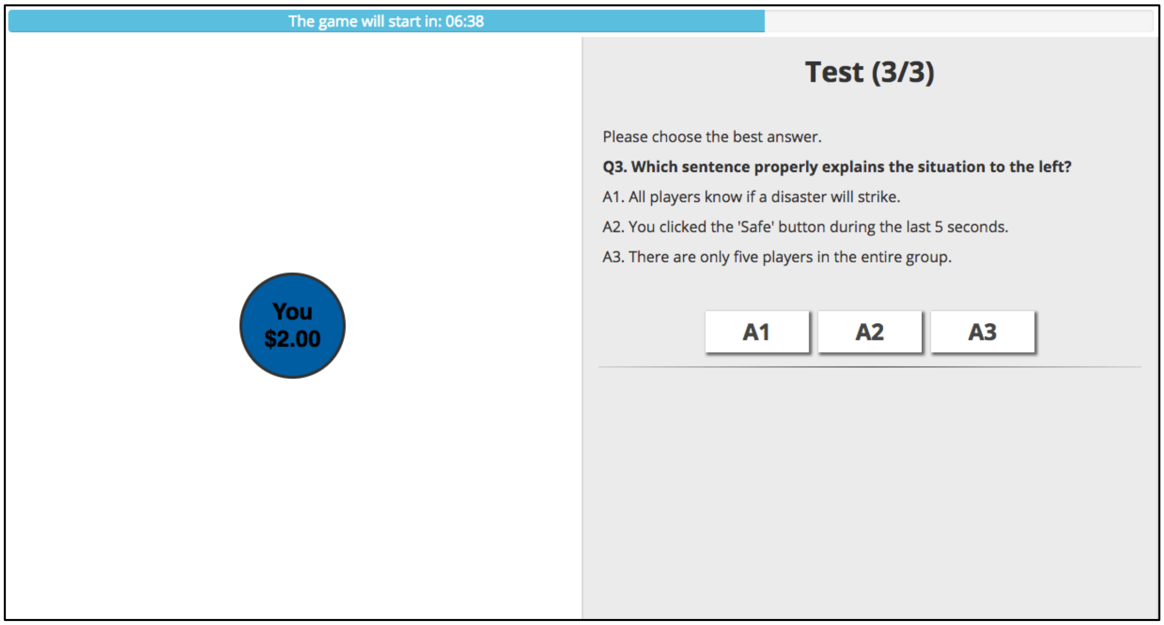
The game will start in: 06:38
You
$2.00
Test (3/3)
Please choose the best answer.
Q3. Which sentence properly explains the situation to the left?
A1. All players know if a disaster will strike.
A2. You clicked the 'Safe' button during the last 5 seconds.
A3. There are only five players in the entire group.
A1
A2
A3

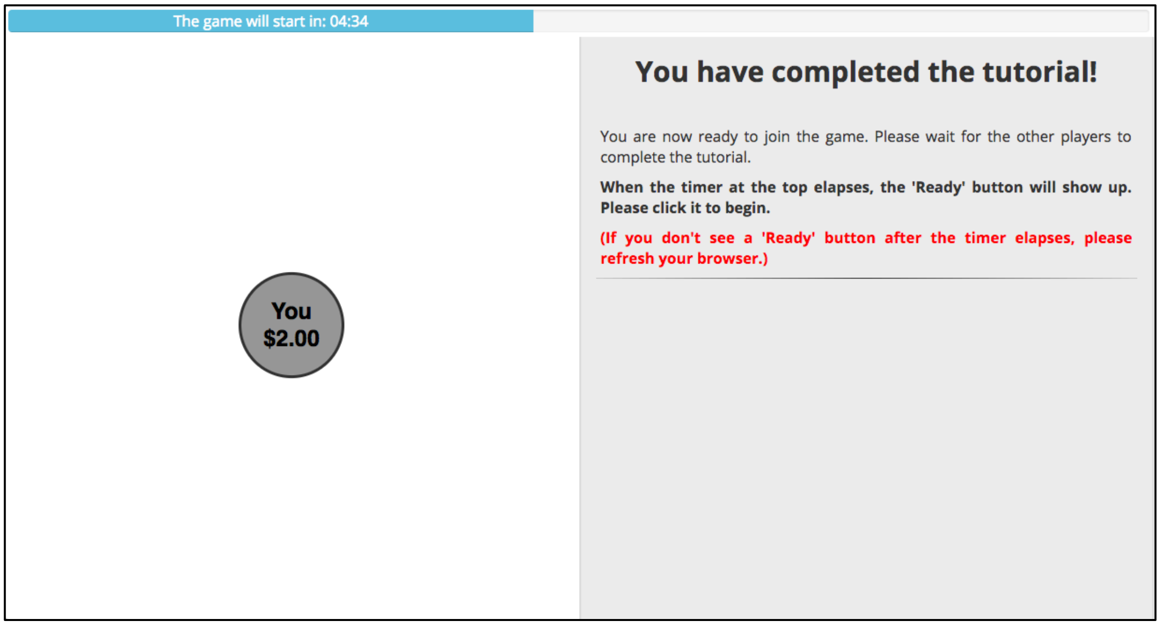

The game will start in: 04:34
You have completed the tutorial!
You are now ready to join the game. Please wait for the other players to complete the tutorial.
When the timer at the top elapses, the 'Ready' button will show up. Please click it to begin.
(If you don't see a 'Ready' button after the timer elapses, please refresh your browser.)
You
$2.00

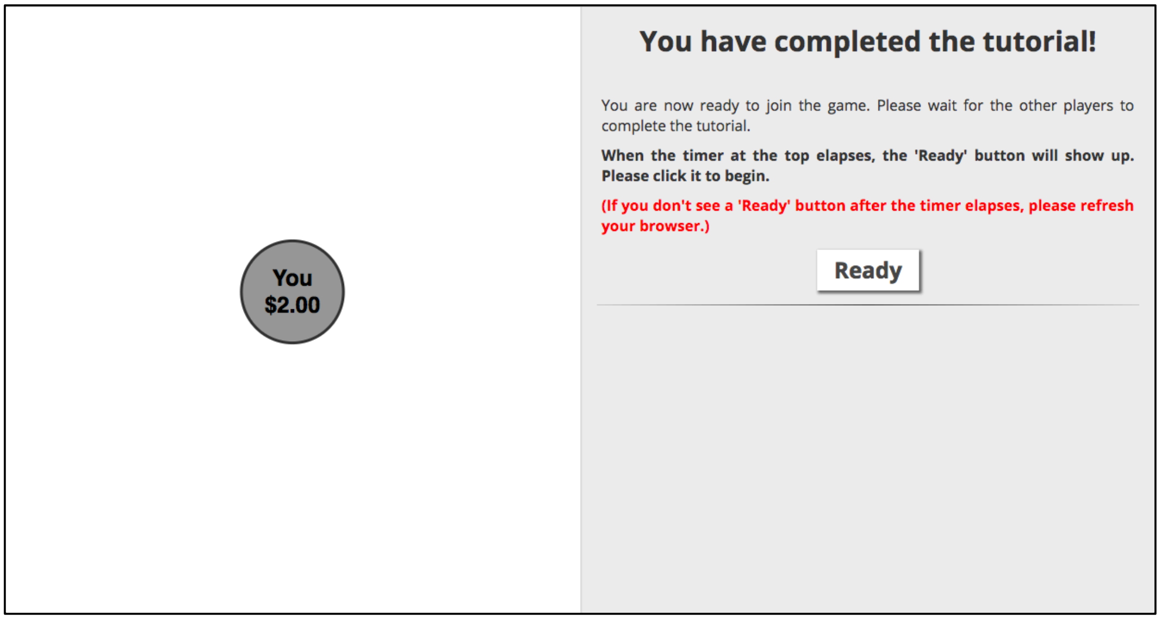

You have completed the tutorial!
You are now ready to join the game. Please wait for the other players to complete the tutorial.
When the timer at the top elapses, the 'Ready' button will show up. Please click it to begin.
(If you don't see a 'Ready' button after the timer elapses, please refresh your browser.)
Ready
You
$2.00

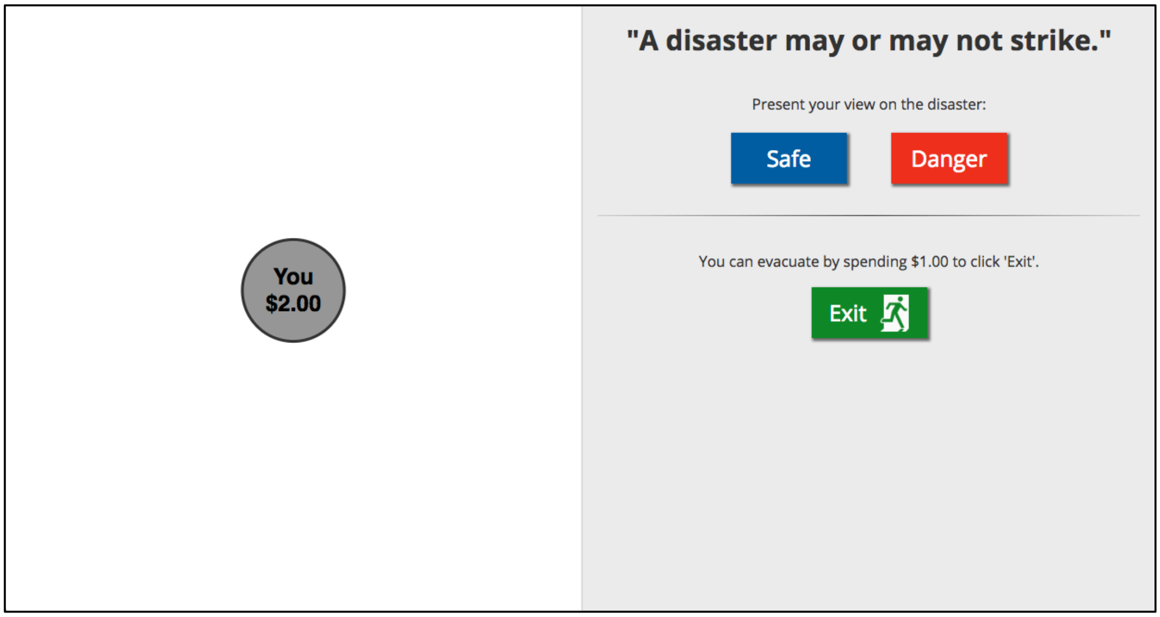
You
$2.00
"A disaster may or may not strike."
Present your view on the disaster:
Safe
Danger
You can evacuate by spending $1.00 to click 'Exit'.
Exit

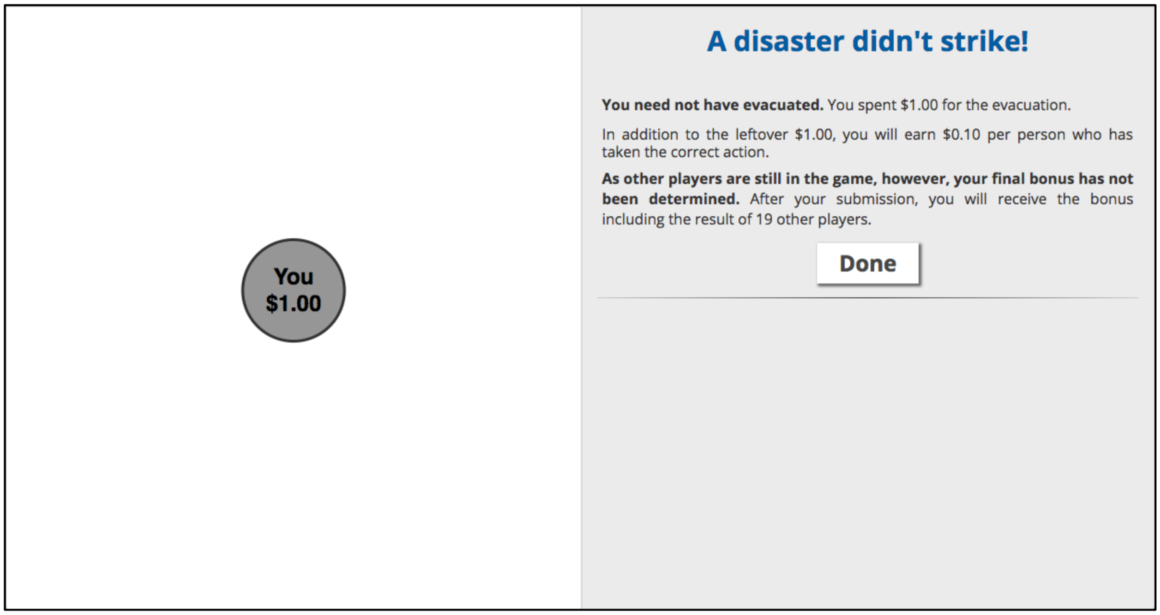
You
$1.00
A disaster didn't strike!
You need not have evacuated. You spent $1.00 for the evacuation.
In addition to the leftover $1.00, you will earn $0.10 per person who has taken the correct action.
As other players are still in the game, however, your final bonus has not been determined. After your submission, you will receive the bonus including the result of 19 other players.
Done

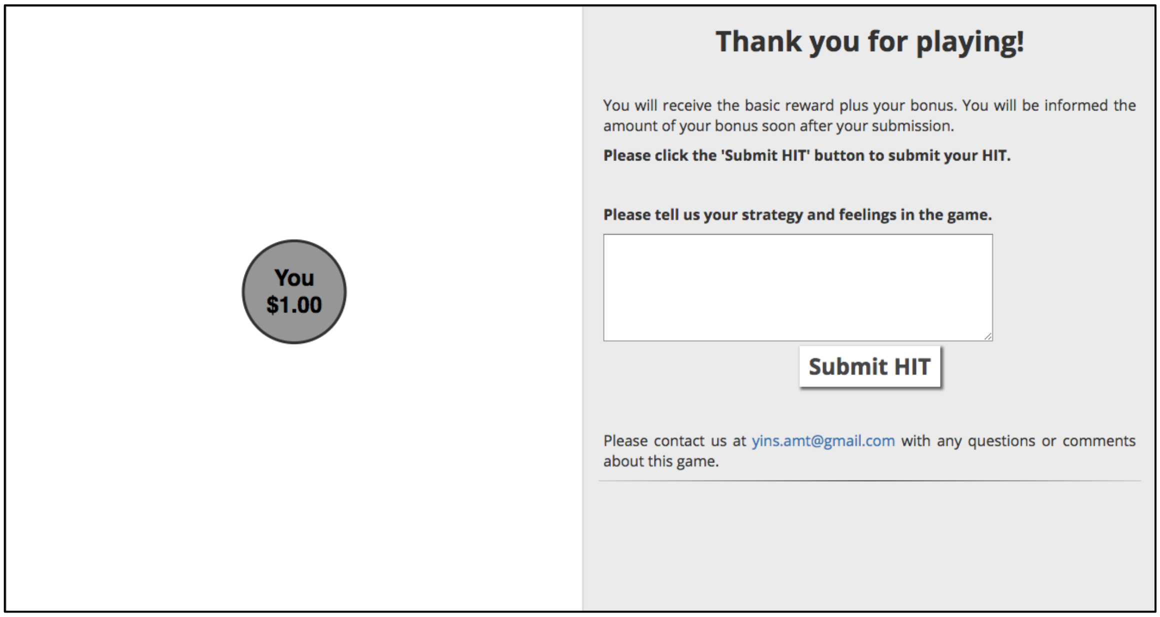
You
$1.00
Thank you for playing!
You will receive the basic reward plus your bonus. You will be informed the amount of your bonus soon after your submission.
Please click the 'Submit HIT' button to submit your HIT.
Please tell us your strategy and feelings in the game.
Submit HIT
Please contact us at yins.amt@gmail.com with any questions or comments about this game.

Modeling of expected utility for sequential decision-making

In the gameplay, as in an actual time-critical situation, players need to make a decision as time advances. As described above, they are not informed when a disaster will strike or when the uncertain situation will end. Thus, some subjects may make a behavioral decision to maximize their utility in the short time cycle (i.e., "discounting" of future gains). Let us suppose subjects estimate the risk of disaster p and the expected profit $\acute{E}$ of each behavioral choice every Δt. Suppose that a player plays with N other players in total and n other players have not evacuated at some point ($n \leq N$). The other players will spontaneously evacuate at the rate k in Δt ($0 < k < 1$), but in the social network condition, the focal player can reduce the spontaneous evacuation at the rate ε_{safe} in Δt ($0 < \varepsilon_{safe} \leq 1 + k$) by sending a safe signal. The player also increases the evacuation rate by ε_{danger} in Δt by sending a danger signal in the social network condition ($0 < \varepsilon_{danger} \leq 1 - k$). The expected profit of each behavior's choice of the player in Δt is:

$$\acute{E}_{Evacuate} = p\big(1.0 + 0.1(N - (1-k)n)\big) + (1-p)(1.0 + 0.1(1-k)n)$$
$$\acute{E}_{Do\ nothing} = (1-p)(2.0 + 0.1(1-k)n)$$
$$\acute{E}_{Send\ safe} = (1-p)(2.0 + 0.1(1-k+\varepsilon_{safe})n)$$
$$\acute{E}_{Send\ danger} = (1-p)(2.0 + 0.1(1-k-\varepsilon_{danger})n).$$

When Δt is close to 0, the chance of a disaster p becomes 0, so that

$$\lim_{\Delta t \to 0} \acute{E}_{Evacuate} = 1.0 + 0.1(1-k)n$$
$$\lim_{\Delta t \to 0} \acute{E}_{Do\ nothing} = 2.0 + 0.1(1-k)n$$
$$\lim_{\Delta t \to 0} \acute{E}_{Send\ safe} = 2.0 + 0.1(1-k+\varepsilon_{safe})n$$
$$\lim_{\Delta t \to 0} \acute{E}_{Send\ danger} = 2.0 + 0.1(1-k-\varepsilon_{danger})n$$

where $0 \leq 1 - k - \varepsilon_{danger} < 1 - k < 1 - k + \varepsilon_{safe} \leq 1$. By making the time cycle Δt shorter, the expected profit of sending a safe signal is the highest among the player's behavior choices in the social network condition. That is, if some players select their behavior with shorter-sighted risk and utility calculation, they are more likely to send a safe signal to their neighbors. In the independent condition, players only choose whether to evacuate. And sequential decision-making can make staying appear more appealing than evacuating.

It is important to note that we do not assume that *every* player follows this behavioral principle. Rather, we show the possibility that *some* players can send a safe signal even without any signal exposure because of their sequential, myopic decision-making. But even if few players take such an ad-hoc approach, they can ignite the spread of safe signals in a social network.

Analysis of supplementary sessions with information availability after evacuation

In the supplementary experiments, involving *N*=1,280 subjects in 64 groups, we also manipulated the additional information that was apparent from evacuated subjects to their neighbors (figure S7A). The main experiment setting simulates the situation of the people who simply lose communication with each other during evacuation. That is, once subjects chose to evacuate, they could not use the communication buttons, but their node was left in a network (set to a gray color). In particular, the neighbors of evacuated subjects were not informed of their evacuation (they simply observed that the departed node communicated neither safe nor danger signals). In the additional two conditions, in terms of information availability, evacuated subjects could send additional signals to their neighbors in a different way. In the "continuous communication" condition, subjects could use the communication buttons even *after* they evacuated. As subjects did not need to refrain from evacuation in order to continue to send signals, there was no dilemma between self-preservation and altruism in this condition. In the "visible evacuation" condition, the nodes of evacuated subjects were removed in their neighbors' network diagram. In contrast to other conditions, subjects could see what neighbors *did* in addition to what they *signaled* (about their sense of whether a disasters was about to strike or not).

The supplementary communication environments work differently for different network topologies (figure S7A). In random-regular networks of all sizes, either allowing evacuated subjects to communicate (continuous signaling) or making neighbor's evacuation visible (visible evacuation) results in significantly higher level of successful evacuation compared to when evacuees stops communicating as in the basic experiments ($P = 0.02$ for the post-evacuation signal condition, and $P < 0.01$ for the visible evacuation condition; long-rank test compared to the basic connectivity condition). On the other hand, in random-regular networks, the additional connectivity settings do *not* affect the level of erroneous evacuation when there is no disaster ($P = 0.28$ for the post-evacuation signal condition, and $P = 0.48$ for the visible evacuation condition).

In contrast, in ring-lattice networks (which have high transitivity), the communication environments have different effects. Allowing post-evacuation signals or making neighbor's evacuation visible does *not* improve the level of successful evacuation in circumstances with a disaster ($P = 0.43$ for the post-evacuation signal condition, and $P = 0.85$ for the visible evacuation condition), but significantly increases false evacuations in circumstances without a disaster ($P = 0.03$ for the continuous communication condition, and $P = 0.02$ for the visible evacuation condition).

As a result, either continuous signaling or visible evacuation closes the gap of overall accuracy between random networks and ring-lattice networks (accuracy at 75s = 0. 581 versus 0.562 in the post-evacuation signal condition; 0.597 versus 0.581 in the visible evacuation condition). There is no significant difference in effectiveness between the additional connectivity settings. In short, the additional signals from evacuated subjects increase successful evacuations from disaster in random networks; and they increase needless evacuations in cases of no disaster in ring-lattice networks. Even in situations that allow the post-evacuation communication or that make evacuation visible, subjects who exchange information in a network are, overall, less likely to evacuate from a disaster, compared to when they make a decision alone.

One of the reasons that the continuous signals do not promote necessary evacuations in ring-lattice networks is an opposing effect of social reinforcement. In random-regular networks, the post-evacuation signals shift the peak of the exposure histogram to having fewer safe and more danger signals. The change of signal exposure increases successful evacuation. In ring-lattice networks, however, the peak does not shift, and the exposure distribution becomes polarized with post-evacuation signals. As a result, the successful evacuation fraction does not improve. The high network transitivity of the ring-lattice network also creates cliques of reassuring subjects with (false) safe signals. True warnings cannot break into the safe-signal clique because the spread of warnings requires more warnings than safe signals (Fig. 4), but this rarely happens at the boundary between safe-signal and danger-signal cliques (see the exposure pattern in the ring-lattice network. The visible evacuation condition has similar pattern as the post-evacuation signal condition.

We also find a difference in effectiveness between simply sharing a warning and showing actual evacuation using the behavioral model with time-varying coefficients. The overall effect on a person's evacuation does not differ between receiving warnings or witnessing evacuation behaviors; however, while the effect of warning does not vary with time, evacuation signals lose their effectiveness as the session progresses. As the game progresses, further evacuees in the system have less impact on the stayers' likelihood of evacuating. On the other hand, evacuation signals enhance the effects of both reassurance and warning later in the session. That is, by making evacuation visible, neighbor's evacuation dramatically increases the risk of evacuation for a short time; after the passage of this initial impact, subjects necessarily rely on fewer remaining, non-evacuated neighbors and they appear to increase the weight they place on information from them.

Analysis of supplementary sessions with other network structures

In the main experiments, subjects were structurally equivalent except for the geodesic distance from an informant. We tested two further types of heterogeneous network structure in additional supplementary experiments involving groups of 20 subjects (figure. S7B), in *N*=420 subjects in 21 groups. In these further experiments, we created ring-lattice networks with shortcuts; these networks, known as "small-world" networks, were created by rewiring an opposite link pair of a ring lattice. The others were standard random networks generated by Erdős-Rényi model with the same number of links as the other networks; these random networks had non-uniform local connections (i.e., subjects did not always have four neighbors). We found that the shortcuts (also known as weak ties or bridges) did *not* facilitate successful evacuation from a disaster; rather, they suppressed evacuation behavior, compared to ring-lattice networks without shortcuts (figure. S7B). On the other hand, subjects in random networks evacuated at the same level of those in the independent condition in 75 seconds (figure. S7B). Although the heterogeneous degree distribution can facilitate the spread of accurate information in the time-critical situation, the collective dynamics heavily depend on the handling of information by high-degree subjects.
